# Phosphorylation of H3.3 at Serine 31 acts as a switch of nucleosome dynamics for transcription

**DOI:** 10.1093/nar/gkaf891

**Published:** 2025-09-12

**Authors:** Jingzhe Ma, Junran Zhang, Xue Xiao, Li Gao, Hang Zhou, Dengyu Ji, Ying Zhang, Guohong Li, Juan Yu, Ping Chen, Wei Li

**Affiliations:** Department of Immunology, School of Basic Medical Sciences, Laboratory for Clinical Medicine, Capital Medical University, Beijing 100069, China; Department of Immunology, School of Basic Medical Sciences, Laboratory for Clinical Medicine, Capital Medical University, Beijing 100069, China; State Key Laboratory of Epigenetic Regulation and Intervention, Institute of Biophysics, Chinese Academy of Sciences, Beijing 100101, China; Department of Immunology, School of Basic Medical Sciences, Laboratory for Clinical Medicine, Capital Medical University, Beijing 100069, China; Department of Immunology, School of Basic Medical Sciences, Laboratory for Clinical Medicine, Capital Medical University, Beijing 100069, China; Department of Immunology, School of Basic Medical Sciences, Laboratory for Clinical Medicine, Capital Medical University, Beijing 100069, China; Department of Immunology, School of Basic Medical Sciences, Laboratory for Clinical Medicine, Capital Medical University, Beijing 100069, China; State Key Laboratory of Epigenetic Regulation and Intervention, Institute of Biophysics, Chinese Academy of Sciences, Beijing 100101, China; New Cornerstone Science Laboratory. State Key Laboratory of Metabolism and Regulation in Complex Organisms, Frontier Science Center for Immunology and Metabolism, Hubei Key Laboratory of Cell Homeostasis, College of Life Sciences, Taikang Center for Life and Medical Sciences, Wuhan University, Wuhan 430072, China; National Laboratory of Biomacromolecules, CAS Center for Excellence in Biomacromolecules, Institute of Biophysics, Chinese Academy of Sciences, Beijing 100101, China; University of Chinese Academy of Sciences, College of Life Sciences, Beijing 100049, China; State Key Laboratory of Epigenetic Regulation and Intervention, Institute of Biophysics, Chinese Academy of Sciences, Beijing 100101, China; Department of Immunology, School of Basic Medical Sciences, Laboratory for Clinical Medicine, Capital Medical University, Beijing 100069, China; State Key Laboratory of Epigenetic Regulation and Intervention, Institute of Biophysics, Chinese Academy of Sciences, Beijing 100101, China

## Abstract

The incorporation of histone variant H3.3 into the genome plays a critical role in regulating gene transcription, genomic stability, and mitosis progression. However, the precise mechanisms underlying the influence of H3.3 on nucleosome stability and dynamics remain poorly understood. In this study, we demonstrate that while the incorporation of H3.3 into nucleosomes does not significantly alter their stability, it enhances the maintenance of nucleosome integrity. Notably, H3.3 recruits the FACT complexes more efficiently than the canonical H3, counteracting FACT’s destabilizing effect on nucleosomes. The binding of FACT to H3.3-nucleosome further stabilizes the nucleosome structure, which can be reversed by phosphorylation at Serine 31 (H3.3S31ph). Through genome-wide analyses, we show that the deposition of H3.3 and its phosphorylation at Ser31 dynamically modulate the nucleosome states, influencing FACT binding and regulating the transcriptional responses in macrophages upon stimulation. The selective phosphorylation at H3.3S31 functions as a pivotal switch, transforming the H3.3-nucleosome from a stable, maintenance-oriented state to a more dynamic, active configuration. This molecular switch enables a rapid response to environmental stimuli, thereby facilitating transcriptional activation. Our findings provide new mechanistic insights into how H3.3 and its Ser31 phosphorylation modulate nucleosome dynamics and transcriptional response, with significant implications for immune response pathways in macrophages.

## Graphical Abstract

**Graphical Abstract:**
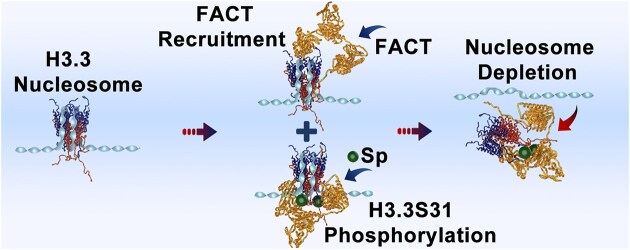

## Introduction

Nucleosome core particle (NCP), the fundamental unit of eukaryotic chromatin, consists of an octamer of histone proteins wrapped by 147 bp of DNA in a left-handed supercoil. It serves as a primary barrier to the access of DNA by various DNA-processing machineries, thereby playing a critical role in almost all DNA-related biological processes [1]. Nucleosome properties and dynamics are central to modulating the accessibility of DNA *cis-*regulatory elements for gene transcription, which is intricately controlled by a variety of distinct epigenetic mechanisms, including DNA methylation, histone variants, and histone post-translational modifications (PTMs) [2]. The histone octamer comprises two copies each of the core histones H2A, H2B, H3, and H4, which form an (H3-H4)_2_ tetramer flanked by two dimers of H2A-H2B. Histone H3 exists in four main variants in metazoans: H3.1, H3.2, H3.3, and centromere-specific variant CENP-A. H3.1 and H3.2, collectively referred to as canonical H3, are encoded by a gene cluster, expressed during the S phase, and incorporated into chromatin during DNA replication [3, 4]. H3.3, one of the most conserved histone variants across eukaryotes, is encoded by two distinct genes, *H3f3a* and *H3f3b*. It is constitutively expressed throughout the whole cell cycle independently of DNA replication [5, 6]. It is noteworthy that in certain simple eukaryotes, such as *Saccharomyces cerevisiae*, a single non-centromeric H3 gene encodes an H3.3-like protein. This suggests that H3.3 is more conserved and evolutionarily ancient than its canonical replicative counterparts.

The incorporation of distinct histone variants has been characterized as one of the most crucial players for gene regulation by modulating the nucleosome turnover and chromatin states or influencing the interactions with chromatin regulators at specific genomic loci. H3.3 is primarily recognized as a marker of transcriptionally active genes, but is also involved in the formation of telomeric and pericentric heterochromatin [7–10]. H3.3 is incorporated into distinct chromatin regions in a DNA replication-independent manner via various mechanisms. The HIRA (histone cell cycle regulator) complex accounts for the assembly of H3.3 at euchromatin regions, while DAXX (death-domain associated protein) is responsible for the deposition of H3.3 at telomeric and pericentric heterochromatin, as well as at endogenous retroviral elements and imprinted loci, in collaboration with ATRX (α-thalassemia X-linked mental retardation protein) [10–15].

As compared to canonical H3.2, H3.3 differs by four specific amino acids: residue 31 (Ser versus Ala) in the N-terminal tail and residues 87 (Ala versus Ser), 89 (Ile versus Val), and 90 (Gly versus Met) in the histone fold domain. Previous studies have shown that these subtle changes in residues contribute to the unique properties and genomic localization of the H3.3 variant. The three specific residues A87, I89, and G90, known as the AIG motif in the histone fold domain, confer an H3.3-specific interaction with DNA and its deposition by histone chaperones DAXX and the HIRA complex [16–19]. Although the unique residue Ser31 in the N-terminal tail does not affect H3.3 deposition, it can be phosphorylated to play an important regulatory role [20–24]. Phosphorylation at Ser31 in H3.3 (H3.3S31ph) was initially identified during metaphase by the mitotic checkpoint kinase CHK1 and the Aurora B to ensure proper chromosome condensation [24]. In macrophages, H3.3S31ph enables rapid transcriptional activation at stimulation-induced genes by ejecting ZMYND11 and recruiting SETD2 [20]. In addition, H3.3S31ph promotes p300-dependent acetylation, particularly at enhancer regions, to drive transcriptional reprogramming during differentiation in mouse embryonic stem cells (mESCs), and plays an essential role in early *Xenopus* embryonic development during gastrulation [21, 22]. Most of these regulatory functions of H3.3S31ph are closely linked to the modifications of neighboring residues, such as H3K36me3 and H3K27me3/ac. Despite the structural similarity between nucleosomes containing canonical H3 and those containing H3.3 [25], how H3.3 and its unique residue Ser31 directly modulate the nucleosome dynamics, and how the mechanisms contribute to gene regulation and biological functions, remain to be elucidated.

In this work, we investigated the direct role of variant H3.3 and its specific residue Ser31 in regulating nucleosome dynamics and their biological functions in the stimulation-induced transcription and macrophage response. Our *in vitro* single-molecule experimental analyses revealed that H3.3 promotes the reassembly of nucleosomes at low force after their mechanical disruption at high force. H3.3 incorporation enhances the maintenance of nucleosome integrity, but does not affect nucleosome mechanical stability. Furthermore, as the FACT (Facilitates Chromatin Transcription) complex is highly co-colocalized with H3.3 at various genomic loci [26], we demonstrated that the FACT complex preferentially binds to H3.3-nucleosomes and further enhances the sturdy property of H3.3-nucleosomes. Significantly, phosphorylation of H3.3S31, but not its point mutation to alanine (S31A) or to phospho-mimicking glutamic acid (S31E), reverses the function of FACT in maintaining nucleosome integrity. H3.3S31 phosphorylation enables nucleosomes to transition from a stable, maintenance-oriented state to a dynamic, active state in the presence of FACT. Genome-wide analyses further revealed that the dynamic phosphorylation of H3.3S31 modulates nucleosome states to facilitate rapid stimulus-induced transcription in macrophages. These findings provide mechanistic insights into the regulation of H3.3 and its S31 phosphorylation on the nucleosomal intrinsic dynamics in transcriptional regulation and the immune response of macrophages.

## Materials and methods

### Protein purification

The recombinant histones H2A, H2B, H3, H4, and H3.3 were purified as previously described [27]. Briefly, all the histone genes were cloned into the pET3a histone expression plasmid, transformed into *Escherichia coli* BL21, and purified from inclusion bodies. The cells were cultured at 37°C to reach the optical density at 600 nm values between 0.5 and 0.6, and induced with the addition of 0.5 mM isopropylthio-β-galactoside for 16 h. The modified histone H3.3S31ph was purchased from KS-V Peptide Biological Technology Co., Ltd (Hefei, China), chemically synthesized using standard solid-phase peptide synthesis and peptide fragment ligation reactions [28]. For purification of the recombinant FACT complex, Sf9 cells (1.5–2 × 10^6^/mL) were infected with baculovirus encoding Flag-SPT16 and His_6_-SSRP1 and incubated for 72 h at 27°C. The cells were collected and washed once with pre-cooled PBS and resuspended in the lysis buffer (150 mM NaCl, 20 mM Tris–HCl, pH 8.0, 5% glycerol, 1 mM PMSF). After ultrasonic crushing, the supernatant was collected after centrifugation and incubated with 500 μL anti-Flag M2-agarose (Sigma, A2220) at 4°C for 4 h, and then washed with lysis buffer. The bound FACT proteins were eluted in the presence of 0.2 mg/mL Flag-peptide (Sigma, F4799) and further purified with heparin HP column (GE Healthcare). The purified protein was dialyzed into BC-100 buffer [100 mM NaCl, 10 mM Tris–HCl, pH 8.0, 0.5 mM ethylenediaminetetraacetic acid (EDTA), 20% glycerol, 1 mM DTT, 1 mM PMSF] and stored at −80°C.

### Nucleosome and tetrasome reconstitution

Respective histone octamers, H3-H4 tetramers, and H2A-H2B dimers were reconstituted as previously described [29]. Equimolar ratios of individual histones in unfolding buffer (7 M guanidinium HCl, 20 mM Tris–HCl, pH 7.5, 10 mM DTT) were dialyzed into refolding buffer (2 M NaCl, 10 mM Tris–HCl, pH 7.5, 1 mM EDTA, 5 mM 2-mercaptoethanol) and purified by Superdex 200 column. For the assembly of mono-nucleosome and tetrasome samples, the reconstituted octamers or tetramers were mixed with DNA template in TEN buffer (10 mM Tris–HCl, pH 8.0, 1 mM EDTA, 2 M NaCl), and dialyzed at 4°C with the buffer continuously diluted by TE buffer (10 mM Tris–HCl, pH 8.0, 1 mM EDTA) to 0.6 M NaCl. The samples were collected after final dialysis in HE buffer (10 mM HEPES, pH 8.0, 0.1 mM EDTA) for 4 h. The stoichiometry of octamer or tetramer to DNA template was determined by atomic force microscopy (AFM) and gel shift analysis [30]. For magnetic tweezers analysis, a 409 bp DNA template containing the middle single 601 sequence was prepared by polymerase chain reaction (PCR) from a plasmid using a biotin (bio)-labeled forward primer and a 3-digoxigenin (3dig)-labeled reverse primer. For mono-nucleosome pull-down assay, a biotin-labeled 210 bp DNA containing the middle single 601 sequence was prepared by PCR as described earlier.

### AFM analysis

A newly cleaved mica surface was treated with 20 μL of spermidine solution (10 mM) and incubated at room temperature for 10 min to facilitate surface functionalization. Following incubation, the mica surface was thoroughly rinsed with 200 μL of deionized water (ddH_2_O) for four cycles and briefly dried under a gentle stream of nitrogen gas. Subsequently, 10 μL of nucleosome solution (1 ng/μL) was deposited onto the functionalized mica surface and incubated for 10–15 min to enable adsorption. The surface was then subjected to an additional four rinsing cycles with 200 μL of ddH_2_O and gently dried with nitrogen gas. The resulting samples were characterized using AFM in ScanAsyst mode, utilizing the MultiMode 8 SPM system (Bruker).

### Single-molecule magnetic tweezers analysis

Single-molecule force-extension measurements of single nucleosomes were performed using magnetic tweezers (HiMT, Qihaobio China). For stretching reconstituted nucleosomes, the two ends of the DNA were independently tethered to a glass coverslip via digoxigenin-anti-digoxigenin ligation and to a 2.8 μm Dynabead (M280, Invitrogen Norway) via biotin-streptavidin ligation. The superparamagnetic Dynabeads were subjected to tension generated by the strong magnetic field gradient produced by two antiparallel NdFeB magnets with a gap of 0.5 mm. The exerted tension on Dynabeads is precisely tuned by moving the magnets with a high-resolution translation stage (M126.PD1, PI Germany), enabling the stretching of the DNA molecule within the nucleosome. The real-time three-dimensional position (x, y, z) of the Dynabead under tension was recorded by a CCD camera (MC1362, Mikrotron Germany) through an inverted microscope objective (UPLXAPO60XO NA 1.42, Olympus Japan). The bright-field diffraction patterns of the bead were analyzed and compared at different focal distances, and the three-dimensional bead position was determined using the quadrant interpolation (QI) algorithm implemented in our LabVIEW software [31].

Force calibration was performed using a 10 000 bp DNA tether between the Dynabead and the coverslip, with measurements repeated for 10 independent DNA molecules. For each magnet position, the y-position of the bead was recorded at a sampling frequency of 500 Hz over 5 min. The forces were quantified via power spectral density analysis [32], and the relationship between magnet position and force was well-fitted to a double exponential function [33]. This calibration was subsequently used to determine the forces applied during the force-extension measurements of the nucleosome [34].

For the force-extension measurements, the flow cells, which consist of a bottom functionalized coverslip with a rectangular channel (5 × 50 mm^2^) and an upper coverslip with two holes at each end, were first incubated with anti-digoxigenin (0.1 mg/mL) for 4 h and then passivated with 10 mg/mL bovine serum albumin (BSA) for 4 h. The nucleosome or tetrasome samples were then injected into the flow cells and tethered between the super-paramagnetic beads (M280, Invitrogen Norway) and the bottom coverslip. To trace the structural transition of samples, magnets were moved near to the samples at a rate of 10 μm/s from 0.1 pN to 30 pN, and the dynamic unfolding process was recorded. The force was rapidly reduced to 0.1 pN and maintained for 5 min, after which the stretching cycle was repeated on the same molecule at least three times. To minimize system drift in the magnetic tweezers, we attached 2 μm polystyrene beads (QDSphere, QDSPS1002 USA) to the coverslip by melting the beads at 155°C for 8 min. During measurements, the z-position of the fixed polystyrene beads was continuously tracked, and the piezo stage was adjusted to move the objective accordingly, keeping the reference beads locked in the same focal plane. The three-dimensional data of the beads were recorded as TDMS files. The force-extension curves and statistical measurements were further analyzed using our LabVIEW and MATLAB software.

To trace the real-time deposition of H2A-H2B dimer by FACT, the mono-tetrasomes were anchored in the flow cells, with 100 μL of the reconstituted FACT complex (50 nM) and H2A-H2B dimer (50 nM) injected and incubated for 30 min. The force-extension measurements for the samples were then traced and recorded. The real-time dephosphorylation dynamics of nucleosomes containing phosphorylated H3.3 histones were investigated. Phosphorylated H3.3 nucleosomes were tethered within the magnetic tweezers, and initial force-extension measurements were performed and recorded three times to establish a baseline. Subsequently, 100 μL of phosphatase solution (10 U/μL) prepared in a 10 × MnCl_2_ buffer was introduced into the flow cell, and the dephosphorylation process was immediately monitored at a constant force of 8.6 pN. A dephosphorylation event corresponding to a 20 nm extension, indicative of the formation of the outer DNA wrap, was recorded in real-time for the same nucleosome. Force-extension measurements were then repeated and recorded for the dephosphorylated H3.3 nucleosome to assess structural changes.

### Mono-nucleosome pull-down assay

Ten microliters of streptavidin-agarose beads (Thermo Fisher Scientific, 29200) were added to each reaction and washed three times with pre-cooled PBS, then mixed with 1 mg/mL of BSA in BC-300 buffer (20 mM Tris–HCl, pH 7.5, 10% glycerol, 300 mM NaCl, 0.1% NP-40) and sealed at 4°C for 1 h. One microgram of biotin-labeled mono-nucleosomes was mixed with the beads in BC-300 buffer and incubated with recombinant FACT complex at graded final concentrations (4 nM and 8 nM) overnight at 4°C. The beads were washed with buffer (20 mM Tris–HCl, pH 7.5, 10% glycerol, 300 mM NaCl, 0.5% NP-40) at 4°C for four cycles, with each cycle lasting 10 min. The proteins bound with beads were eluted with buffer (50 mM Tris, pH 6.8, 2% SDS, 0.1% bromophenol blue, 10% glycerol, 1% beta-mercaptoethanol) and analyzed by western blot.

### Western blot analysis

The denatured proteins were separated by sodium dodecyl sulfate–polyacrylamide gel electrophoresis (SDS–PAGE), transferred to a PVDF membrane (Millipore, ISEQ00010), and blocked with 5% skim milk at room temperature for 1 h. The membrane was incubated with the corresponding primary antibody at 4°C overnight, then washed with TBST three times, with each time lasting 5 min. The membrane was then incubated with the corresponding HRP-conjugated secondary antibody at room temperature for 1 h. The reaction was visualized using Pierce Western ECL substrate (Thermo Fisher Scientific, 32109) and detected by exposure to the gel documentation system. The antibodies used include anti-SPT16 antibodies (Cell Signaling Technology, 12191, 1:2000), anti-SSRP1 antibodies (Biolegend, 609702, 1:2000), anti-HA antibodies (Abcam, ab9110, 1:4000), anti-H3 antibodies (ABclonal, A2348, 1:10 000), goat anti-rabbit second antibody (Zhongshan Golden Bridge Bio-technology, ZB2301, 1:10 000), and goat anti-mouse second antibody (Zhongshan Golden Bridge Bio-technology, ZB-2305, 1:10 000).

### Cell culture and siRNA transfection

RAW264.7 cells were cultured in Dulbecco’s modified Eagle’s medium (DMEM) (Gibco, C11995500BT) with 10% inactivated fetal bovine serum (FBS, Hyclone, SH30071), 1% penicillin, and 1% streptomycin in an incubator at 37°C and 5% carbon dioxide. For macrophage activation, the cells were stimulated with 50 ng/mL of lipopolysaccharide (LPS, InvivoGen, tlrl-3pelps) and 100 ng/mL of interferon-gamma (IFN-γ, BioLegend, 575304) for 30 min or 4 h. The cells were treated with the inhibitor IKK-16 (Selleckchem, S2882) at a concentration of 6 μM for 2 h prior to stimulation. The control group used the same volume of dimethyl sulfoxide (Sigma, A32957) as IKK-16. CRISPR/Cas9-mediated genome editing techniques were used to generate the H3.3 double knockout (DKO) cell lines by targeting two coding genes of H3.3, with *H3f3a* first targeted and then *H3f3b*. The gRNAs as follows were cloned into PX260 (modified to contain the guide sequence insertion site of PX330) and sorted for puromycin (InvivoGen, ant-pr-1) after transfection. Cells were sorted into single-cell clones. PCR and sequencing were used to detect positive clones. SPT16 expression was knocked down in RAW264.7 cells, with the siRNA transfected using Lipofectamine 3000 (Thermo Fisher Scientific, L3000015) according to the manufacturer’s protocol, and cells were collected after being cultured for 36 h for reverse transcription quantitative PCR (RT-qPCR) or western blot detection.

The gRNAs for *H3f3a*

forward primers: caccTAGAAATACCTGTAACGATG

reverse primer: aaacCATCGTTACAGGTATTTCTA;

The gRNAs for *H3f3b*

forward primer: caccGAAAGCCCCCCGCAAACAGC

reverse primer: aaacGCTGTTTGCGGGGGGCTTTC

The sequence of SPT16 siRNA oligos is:

sense 5′GGUCCAGCCACUAUUCUUATT-3′

antisense 5′-UAAGAAUAGUGGCUGGACCTT-3′

### Lentiviral transduction

HEK 293T cells were cultured in DMEM medium (Gibco, C11995500BT) with 10% fetal bovine serum (FBS, Hyclone, SH30071) in an incubator containing 5% carbon dioxide at 37°C. Cell cultures were periodically tested for mycoplasma contamination. The cells were transfected with a third-generation lentiviral vector [containing H3.3 transgene, including wild-type (WT) H3.3 and the mutant H3.3S31A], packaging vectors (pREV, pMD2G, and pVSVG), and a calcium phosphate transfection kit (Invitrogen) to generate lentivirus, which was then used to transduce H3.3 DKO RAW264.7 cells. After 72 h of culture, puromycin (InvivoGen, ant-pr-1) was added to screen for overexpressing cells. The overexpression level was evaluated by western blot.

### RNA extraction and RT-qPCR analysis

Cells were collected, and RNA was extracted using an RNA extraction kit (Vazyme, R401-01). The extracted RNA was reverse-transcribed into complementary DNA (cDNA) using a cDNA reverse transcription kit (Vazyme, R223-01). qPCR was performed with SYBR fluorescent dye and normalized to *β-actin*. The primer pairs used for the qPCR experiments were the following:

*β* *-actin*: sense 5′-GTGCTATGTTGCTCTAGACTTCG-3′;

antisense 5′-ATGCCACAGGATTCCATACC-3′;

*SPT16*: sense 5′-ATCAATGCTATTCCTGTTGCCTCTC-3′;

antisense 5′-CCGTCCTCAGCGTCACTCC-3′;

*Runx1*: sense 5′-CACCCAGCGACACCCATTTCAC-3′;

antisense 5′-CGGAGATGGACGGCAGAGTAGG-3′;

*Usp25*: sense 5′-ACTGGAAAGAAGAAACGCTCC-3′;

antisense 5′-TGGGCAACTTTCATTATGTTGTGA-3′;

*Trem3*: sense 5′-GTCACCGCTGCTGCTATGGC-3′;

antisense 5′-GGTCAGGTTCTCGCCCTCCAG-3′

### Cell phagocytosis assay

The cells were inoculated into six-well plates with 5 × 10^5^ cells per well. After the cells were attached to the wall, the working concentration of PE-labeled beads (Thermo Fisher Scientific, F13083) was added to the well. After 24 h of cultivation, cells were collected and resuspended with 500 μL PBS. The phagocytic active (PE^+^) macrophage cells were measured by flow cytometry from 200 000 cells in each group to calculate the proportion of PE^+^ cells.

### Cell migration assays

Transwell Permeable Supports, containing a 6.5 mm insert and an 8 μm polycarbonate membrane at the bottom of the upper chamber (Corning, 3422), were used for migration experiments. The collected cells were washed and resuspended in serum-free medium, and counted by cell counter. Each upper chamber was loaded with 3 × 10^4^ cells with a total volume of 100 μL, and the lower chamber was filled with a complete medium containing 20% inactivated FBS of 700 μL. After 48 h of cell culture, the media from the upper and lower chambers were removed, and 1 mL of PBS was added to clean the cells. The migrating cells adhering to the bottom surface of the chamber membrane were stained with 0.2% crystal purple and observed under a microscope. Images of three different regions were captured on each membrane, and the number of migrating cells was calculated using the ImageJ image processing program.

### ChIP-seq analysis

RAW264.7 cells in the resting state or cells in the stimulated state after stimulation with 100 ng/mL LPS and 50 ng/mL IFN-γ were collected and crosslinked in serum-free DMEM medium containing 1% formaldehyde at room temperature for 10 min, and then 0.125 M glycine was added to terminate the crosslinking. After centrifugation, NLB buffer (50 mM Tris–HCl, pH 8.0, 10 mM EDTA, 1% SDS, protease inhibitors) was added to lyse nuclei, and then chromatin was broken into fragments of 200–350 bp by ultrasound. The corresponding antibody was first bound to protein A/G-dynamic beads (Life Technology, 10 mL) at 4°C for 6 h and then added to chromatin fragments at 4°C overnight. A consistent quantity of the *Drosophila* reference genome was incorporated into each experimental group. The beads were washed with RIPA-150 (50 mM Tris–HCl, pH 8.0, 150 mM NaCl, 1 mM EDTA, 0.1% SDS, 1% Triton X-100, 0.1% sodium deoxycholate), RIPA-500 (50 mM Tris–HCl, pH 8.0, 0.5 M NaCl, 1 mM EDTA, 0.1% SDS, 1% Triton X-100, 0.1% sodium deoxycholate), RIPA-LiCl (50 mM Tris–HCl, pH 8.0, 1 mM EDTA, 1% NP-40, 0.7% sodium deoxycholate, 0.5 M LiCl), and TE buffer (10 mM Tris–HCl, pH 8.0, 1 mM EDTA) for 5 min each time and were then resuspended with NLB buffer. After decrosslinking at 65°C for 6 h, RNase A and protease K were added to remove RNA and proteins. All ChIP experiments produced paired-end sequencing data, with each fragment measuring 150 bp in length. Prepared the DNA library using the VAHTS Universal DNA Library Prep Kit for Illumina V3 (Vazyme, ND607). Used the Illumina NovaSeq 6000 for high-throughput sequencing. Each sample was performed with two independent biological replicates. FastQC (version 0.12.1) was used to check the data quality after the sequencing data was released. Cleaned the data and aligned it with Mus musculus (mm10) using Cutadapt (version 0.6.10) and Bowtie2 (version 2.5.1). Removed duplicates using Samtools (version 1.17) and mapped the data to call peaks with MACS (version 1.4.2). After mapping, the reads were normalized to the percentage of reference genome reads in the sample. Marked the positions of peaks and annotated peaks by ChIPSeeker (version 1.36.0). Calculated read density and counted reads with Homer (version 4.11). IGV (version 2.18.2) was used to view the sequencing tracks. Performed all analyses profiling using R (http://www.r-project.org/).

### CUT&Tag analysis

The CUT&Tag Assay Kit (Vazyme, TD903) was used for the experiment. The RAW264.7 cells were collected and counted, then the pre-cooled NE buffer was added to resuspend the cells for nuclear extraction, and they were incubated on ice for 10 min. After centrifuging and discarding the supernatant, 1% formaldehyde was added for crosslinking, and glycine was added for termination following the incubation at room temperature for 2 min. The nuclei were resuspended with 100 μL wash buffer, then activated ConA beads were added. The mixture was inverted to mix, incubated at room temperature for 10 min, then placed on a magnetic rack, and the supernatant was discarded. Each sample was supplemented with 50 μL of pre-cooled antibody buffer resuspension nucleus-magnetic beads mixture. Antibodies were added according to the recommended immune concentration and incubated overnight at 4°C. On the second day, the corresponding secondary antibodies were added and incubated for 1 h. pA/G-Tnp was added and incubated for 1 h, then added TTBL to fragment the DNA. Prepared the DNA library using the TruePrep Index Kit V2 for Illumina (Vazyme, TD202). Used the Illumina NovaSeq 6000 for high-throughput sequencing. Each sample was performed with two independent biological replicates. Data analysis was similar to the above ChIP-seq data analysis.

### RNA-seq analysis

RAW264.7 cells were cultured to a density of 5 × 10^6^ cells per well in DMEM medium with 10% FBS, followed by treatment as described in the ChIP-seq analysis protocol. Total RNA was extracted from each sample using an RNA extraction kit. RNA libraries were prepared from 50 ng/μL, 10 μL aliquots of each sample. High-throughput sequencing was conducted on the Illumina NovaSeq 6000 platform. Each sample was subjected to three independent biological replicates. Data quality was assessed with FastQC (version 0.12.1), and sequencing reads were cleaned using Cutadapt (version 0.6.10). Alignment was performed with HISAT2 (version 2.2.1) against the Mus musculus (mm10) reference genome. Duplicate reads were removed using Samtools (version 1.17), and gene-level annotation was conducted with FeatureCounts (version 2.0.6). Each sample contained 33 891 annotated transcripts. Principal component analysis was conducted using DESeq2 (version 1.40.2) and ggplot2 (version 3.5.0) to visualize the data. The gene expression data generated by FeatureCounts (version 2.0.6) was normalized using the DESeq2 package (version 1.40.2) of R (version 4.3.1). The average values of the normalized data were calculated from the three independent biological replicates. To reduce dispersion, the logarithm (base 10) of each data point was calculated after adding 1 to each data point, to avoid issues with calculating the logarithm of zero values in the normalized expression data. Box plots were generated using GraphPad Prism (10.1.2) to compare gene expression across different samples. *P*-values of the statistical tests were calculated using the Wilcoxon matched-pairs signed rank test by dplyr (version 1.1.4) of R (version 4.3.1). Differentially expressed genes were identified by intersecting genes annotated from ChIP-seq with those from RNA sequencing (RNA-seq). Enrichment analyses were performed using the Database for Annotation, Visualization, and Integrated Discovery (DAVID, https://david.ncifcrf.gov/) and Metascape (http://metascape.org/).

### MNase-seq analysis

RAW264.7 cells were cultured to a density of 5 × 10^6^ cells per well in DMEM medium with 10% FBS, following the treatment protocol described in the “ChIP-seq analysis” section. The cells were completely trypsinized and then centrifuged at 1000 rpm for 5 min. Crosslinking was carried out using 1% formaldehyde for 10 min and then neutralized with 0.125 M glycine for 5 min. The *Drosophila* Schneider 2 (S2) cells were prepared using the same method as described for the ChIP-seq analysis earlier. Cells were lysed using Buffer A (10 mM Tris–HCl, pH 7.5, 60 mM KCl, 15 mM NaCl, 3 mM MgCl_2_, 1 mM DTT, protease inhibitors) with 0.5% CA630 (Sigma, I3021), incubated on ice for 5 min, and then quickly centrifuged. Buffer A with 0.1% CA630 was used to resuspend the cells and wash once. For 5 × 10^6^ cells and 5 × 10^5^*Drosophila* Schneider 2 (S2) cells per group, 1 mL of Buffer A with 0.1% CA630 was used, and 0.1 U/mL MNase was added to incubate the mixture for 5 min at 37°C. Then, 2 mM CaCl_2_ was added to digest for 22 min. The digestion was stopped by adding 10 mM EGTA. To reverse crosslinking, 0.35 M NaCl and 1% SDS were added and incubated at 65°C for 6 h. DNA was purified with RNase A and proteinase K, and extracted using phenol-chloroform-isoamyl alcohol extraction. The DNA library was prepared using the VAHTS Universal DNA Library Prep Kit for Illumina V3 (Vazyme, ND607). High-throughput sequencing was performed on the Illumina NovaSeq 6000. Each sample was processed with two independent biological replicates. Data quality was checked using FastQC (version 0.12.1). The data were cleaned and aligned to Mus musculus (mm10) using Cutadapt (version 0.6.10) and Bowtie2 (version 2.5.1). Duplicates were removed using Samtools (version 1.17), and peak calling was performed with MACS (version 1.4.2). After mapping, the reads were normalized to the percentage of reference genome reads in the sample. Nucleosome signal read density was calculated and counted with Homer (version 4.11). All analyses were visualized using ggplot2 (version 3.5.0) using R (http://www.r-project.org/).

### Statistical analysis

All data analyses were conducted using GraphPad Prism (version 10.1.2) and MATLAB (version 2024b). The results of these analyses, such as *P*-values and number of samples, were displayed within the figures or detailed in the associated figure legends. The statistical results were considered significant if the calculated *P*-value was below the threshold of .05.

## Results

### H3.3 preserves nucleosome integrity without altering its mechanical stability

**Figure 1.**
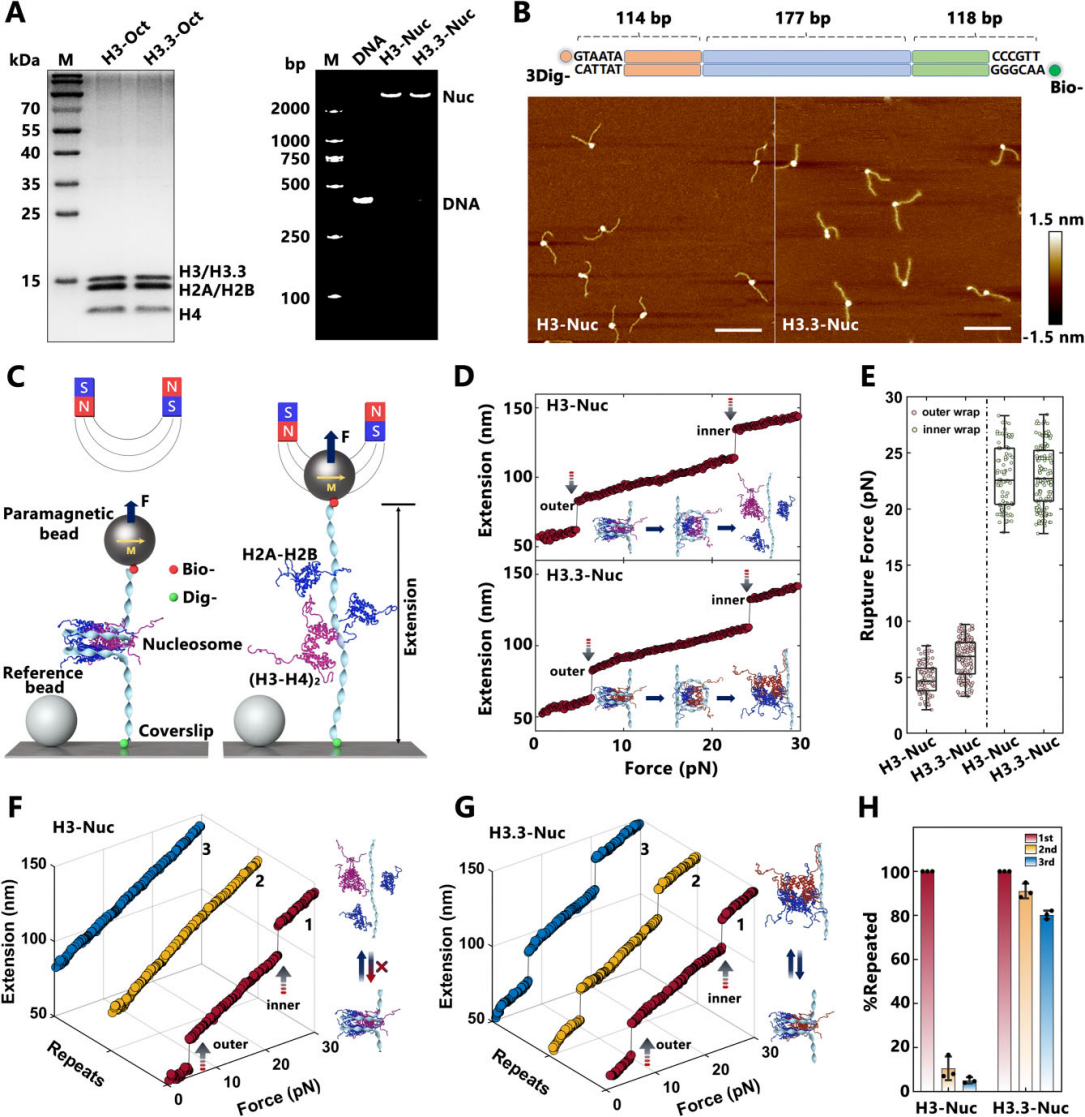
H3.3 maintains nucleosome integrity, but has little effect on nucleosome mechanical stability. (**A**) SDS–PAGE analysis (left) of purified histone octamers with canonical H3 (H3-Oct) and H3.3 (H3.3-Oct), and 1% agarose gel electrophoresis analysis (right) of reconstituted H3-nucleosomes (H3-Nuc) and H3.3-nucleosomes (H3.3-Nuc) for magnetic tweezers analysis, using free DNA template (DNA) as a control. The first lane shows the molecular weight marker (M). (**B**) AFM images of reconstituted H3-Nuc (left) and H3.3-Nuc (right), with the DNA template shown on top. Scale bar: 100 nm. Height: −1.5 to 1.5 nm. (**C**) Schematic representation of nucleosome stretching experiments using magnetic tweezers (not to scale). (**D**) Typical force–extension curves of H3-Nuc (top) and H3.3-Nuc (bottom) measured by magnetic tweezers. (**E**) Statistical analysis of rupture forces for the outer and inner DNA wraps shown in panel (D) (*n* = 98 for H3-Nuc, *n* = 150 for H3.3-Nuc). Box plots display the median (line), interquartile range (IQR, box), data distribution (whiskers), and outliers (points beyond ± 1.5 × IQR). (**F**, **G**) Typical multiple stretching measurements of H3-Nuc and H3.3-Nuc, where each stretching cycle applied a force up to 30 pN. (**H**) The proportion of nucleosomes maintained across three stretching measurements for H3-Nuc and H3.3-Nuc.

To investigate the role of histone variant H3.3 in nucleosome mechanics and structural dynamics, we employed magnetic tweezers to examine its effects on nucleosome stability and folding kinetics through direct single-nucleosome stretching. Mono-nucleosomes containing either canonical H3 or the variant H3.3 were reconstituted *in vitro* on 409 bp DNA containing one Widom 601 nucleosome positioning sequence in the middle and examined by AFM imaging (Fig. 1A and B). Real-time force-extension trajectories of individual nucleosomes were recorded to monitor force-dependent conformational changes (Fig. 1C). When subjected to exerted forces ranging from 0.1 to 30 pN, both H3- and H3.3-containing nucleosomes exhibited a characteristic two-step unfolding process, corresponding to the sequential unraveling of the outer and inner DNA wraps of the nucleosome (Fig. 1D). Statistical analysis of rupture forces revealed no significant differences between the two nucleosome types (Fig. 1E). For H3.3-nucleosomes, the rupture forces for the outer and inner DNA wraps were ∼6.6 pN and 22.9 pN, respectively, comparable to those of canonical H3-nucleosomes. These findings are consistent with previous studies, which demonstrated that H3.3 does not alter the overall mechanical stability of nucleosomes [27, 35].

Interestingly, multiple stretching and relaxing cycles revealed a distinct difference in the ability of H3- and H3.3-containing nucleosomes to maintain structural integrity (Fig. 1F and G). Canonical H3-nucleosomes exhibited the characteristic two-step unfolding behavior only during the first stretching cycle. The stretching behavior in subsequent cycles was consistent with that observed in the naked DNA template (Supplementary Fig. S1), suggesting that canonical H3-nucleosomes fail to reassemble correctly after complete disruption, likely due to the displacement of core histones from the DNA [36]. In contrast, H3.3-nucleosomes consistently retained the two-step unfolding pattern across multiple stretching cycles, indicating that the variant H3.3 promotes the maintenance of nucleosome integrity. The maintenance properties of nucleosome integrity were quantitatively analyzed across the repeated stretching cycles (Fig. 1H). These results demonstrated that while H3.3 does not influence the mechanical stability of nucleosomes, it plays a critical role in preserving their structural integrity during dynamic processes.

### FACT preferentially binds H3.3-nucleosomes to form a sturdy nucleosome state

**Figure 2.**
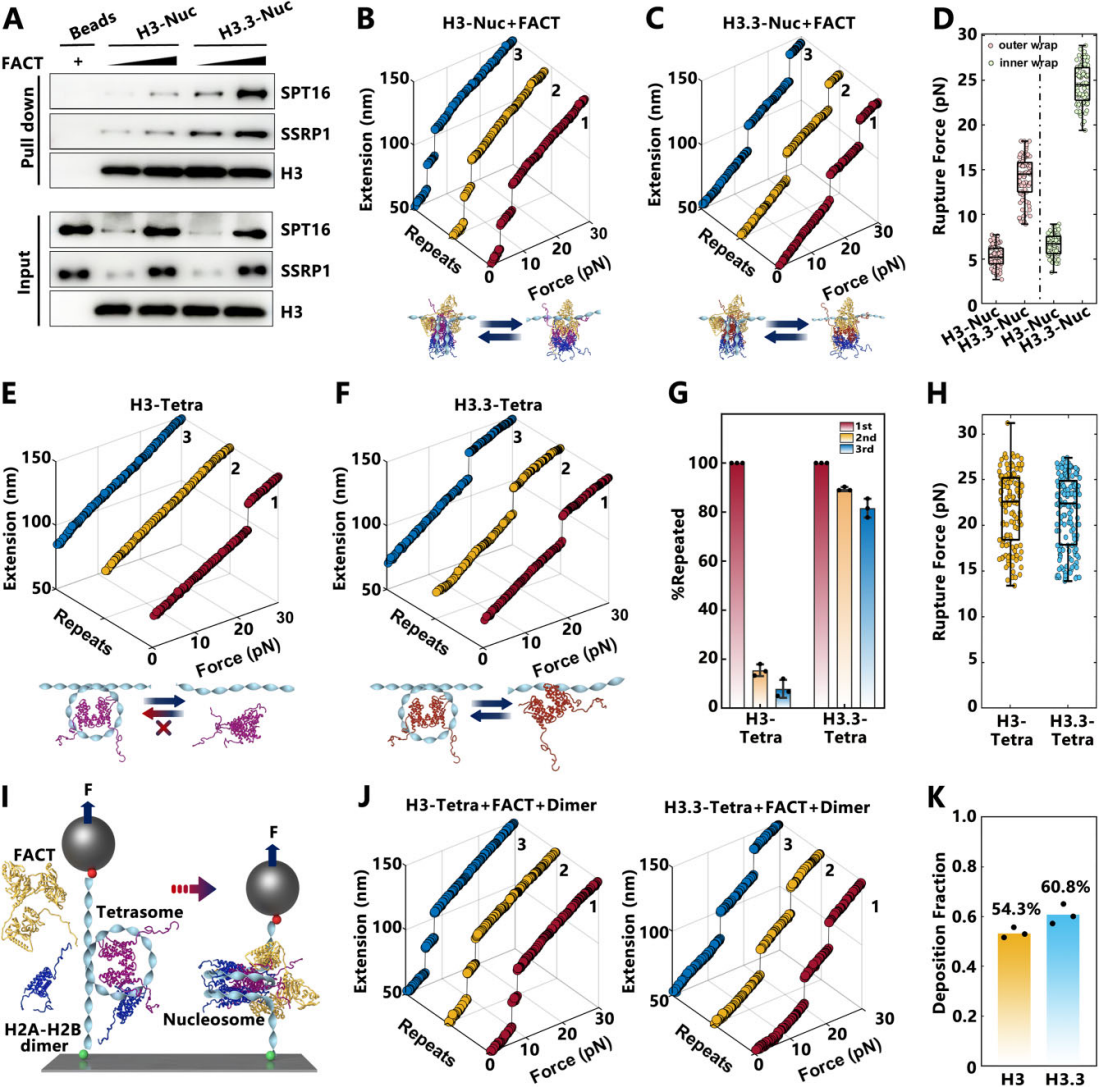
FACT prefers to bind to H3.3-nucleosomes to form a sturdy nucleosome state. (**A**) A mono-nucleosome pull-down assay of H3-nucleosomes (H3-Nuc) and H3.3-nucleosomes (H3.3-Nuc) incubated with the FACT complex (with subunits SSRP1 and SPT16) demonstrated that FACT preferentially binds to H3.3-nucleosomes compared to H3-nucleosomes (*n* = 3). (**B**, **C**) Representative multiple stretching measurements of H3-Nuc and H3.3-Nuc in the presence of FACT. (**D**) Statistical analysis of rupture forces for the outer and inner DNA wraps, as shown in panels (B) and (C) [*n* = 85 for panel (B), *n*= 93 for panel (C)]. (**E**, **F**) Representative multiple stretching measurements of H3-tetrasomes (H3-Tetra) and H3.3-tetrasomes (H3.3-Tetra). (**G**) Proportion of tetrasome maintained across three stretching cycles for H3-Tetra and H3.3-Tetra as shown in panels (E) and (F). (**H**) Statistical analysis of rupture forces for the outer and inner DNA wraps for H3-Tetra and H3.3-Tetra as shown in panels (E) and (F) [*n* = 135 for panel (E), *n* = 161 for panel (F)]. (**I**) Schematic representation of *insitu* nucleosome reconstitution on the tetrasome incubated with FACT and H2A-H2B dimer using magnetic tweezers (not to scale). (**J**) Typical multiple stretching measurements of the H3-Tetra and H3.3-Tetra incubated with FACT and H2A-H2B dimer. (**K**) Quantitative analysis on FACT deposition efficiency for H2A-H2B dimer onto H3-Tetra and H3.3-Tetra.

Previous investigations have shown that the FACT complex highly co-localizes with H3.3 across various genomic loci and promotes the H3.3 deposition at the *white* gene enhancer in *Drosophila* [26, 37]. Given this, we sought to explore how the FACT complex interacts with H3.3 to modulate the nucleosome state and function. Specifically, we examined how the variant H3.3 influences the binding of FACT complex at the nucleosome level. The recombinant FACT complex, consisting of two subunits, SPT16 and SSRP1, was purified (Supplementary Fig. S2A) and incubated with mono-nucleosomes reconstituted with biotinylated DNA and canonical H3 or H3.3 histones. The mono-nucleosome pull-down assay revealed that FACT prefers to bind H3.3-nucleosomes compared to canonical H3-nucleosomes (Fig. 2A).

To further investigate the regulatory role of FACT in the presence of H3.3, we examined the mechanical behavior of nucleosomes upon FACT binding. In the case of canonical H3-nucleosomes incubated with FACT, the nucleosomes were observed to be completely disrupted at low tensions (the rupture forces centered around 6.7 pN), and the structural transitions remained consistent across repeated stretching cycles (Fig. 2B), which aligns with previous findings suggesting that FACT impairs the nucleosome stability and maintains nucleosome integrity simultaneously [38]. Conversely, when H3.3-nucleosomes were incubated with FACT, complete nucleosome disruption occurred only at much higher tensions (the rupture forces centered around 24.5 pN) (Fig. 2C). Statistical analysis of rupture forces for both canonical H3- and H3.3-nucleosomes in the presence of FACT (Fig. 2D) revealed that FACT stabilizes the H3.3-nucleosomes, whereas it destabilizes canonical H3-nucleosomes. Moreover, multiple stretching cycles of the H3.3-nucleosomes incubated with FACT retained a similar force response, indicating that the integrity of these nucleosomes was preserved across multiple cycles (Supplementary Fig. S2B). These findings indicated that FACT preferentially binds to H3.3-nucleosomes and promotes the formation of a stable, resilient nucleosome state, which is crucial for the regulation of chromatin structure during transcriptional processes.

The FACT complex is known as a chaperone of histone H2A-H2B dimers, facilitating their deposition onto the (H3-H4)_2_ tetrasomes (the particles made of DNA wrapped around an H3-H4 tetramer) to form intact nucleosomes [38, 39]. We next investigated how H3.3 affects the H2A-H2B chaperone property of the FACT complex. To address this, we reconstituted mono-(H3-H4)_2_ tetrasomes (referred to as H3-tetrasomes) or mono-(H3.3-H4)_2_ tetrasomes (H3.3-tetrasomes) *in vitro* on the 409 bp DNA fragment with one Widom 601 nucleosome positioning sequence, and subjected these tetrasomes to mechanical analysis using the magnetic tweezers (Fig. 2E and F and Supplementary Fig. S2C). We found that H3.3 does not affect the mechanical stability of tetrasomes, but it does help maintain their integrity (Fig. 2G and H). Both H3- and H3.3-tetrasomes exhibited a one-step transition around 22.5 pN, corresponding to the disruption of the inner nucleosome wrap (Fig. 2H), consistent with previous studies [38]. Intriguingly, the one-step transition is not repeatable for H3-tetrasomes, whereas H3.3-tetrasomes exhibited repeatable transitions, indicating that H3.3 contributes to the maintenance of tetrasome integrity (Fig. 2G). When the H3.3-tetrasomes were incubated with both FACT and H2A-H2B dimers, a repeated two-step structural transition was observed across the multiple stretching cycles, similar to that observed for canonical H3-tetrasomes (Fig. 2I and J). This suggested that FACT can effectively deposit the H2A-H2B dimers onto H3.3-tetrasomes, forming intact nucleosomes, just as it does with canonical H3. Additionally, quantitative analysis of H2A-H2B dimers’ deposition efficiency revealed that 60.8% ± 3.9% (mean ± SD) of H3.3-tetrasomes were successfully assembled into nucleosomes, compared to 54.3% ± 2.9% (mean ± SD) for H3-tetrasomes (Fig. 2K). These results indicated that H3.3 does not substantially affect FACT’s function as an H2A-H2B chaperone, confirming that the variant does not hinder the chromatin assembly process.

Together, these findings demonstrated that the presence of H3.3 enhances the stability and integrity of nucleosomes, a property that is crucial for maintaining chromatin structure during transcriptional regulation. The preferential binding of FACT to H3.3-nucleosomes and its ability to stabilize nucleosomes may provide a mechanistic basis for the role of H3.3 in regulating gene expression and chromatin dynamics.

### H3.3 Serine 31 phosphorylation abrogates FACT’s maintenance function at the nucleosome level

**Figure 3.**
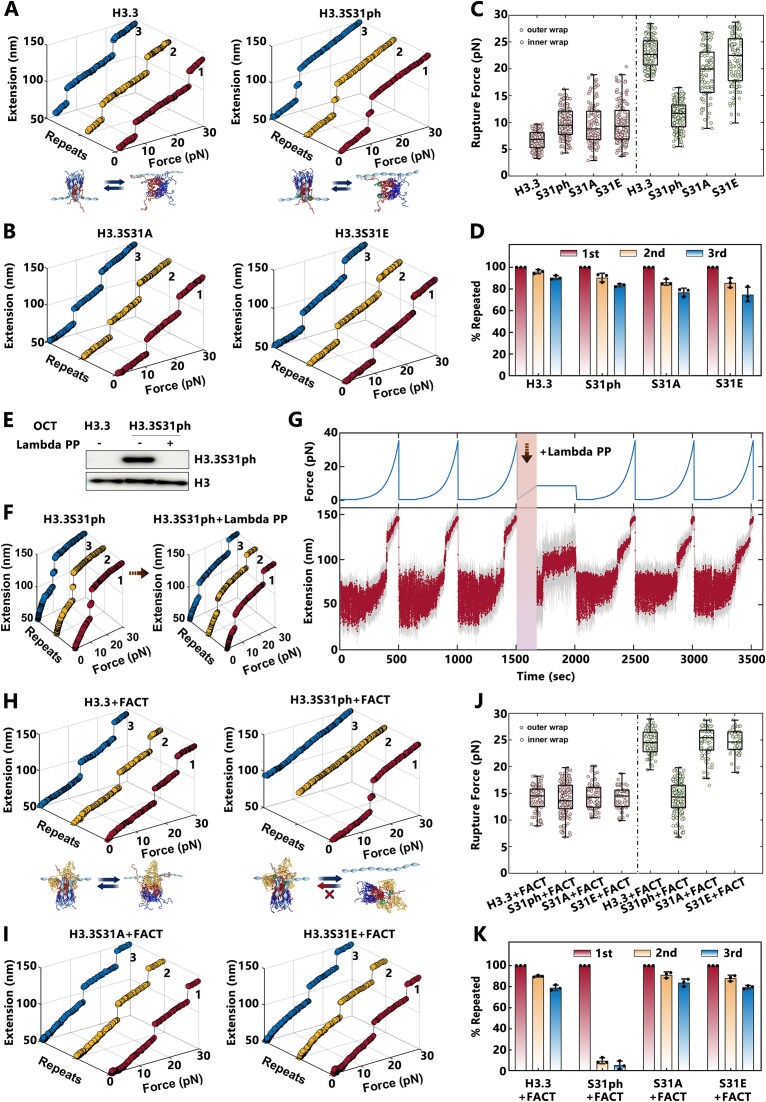
Phosphorylation of H3.3 at Serine 31 (H3.3S31ph) disrupts the maintenance function of FACT at the nucleosome level. (**A**, **B**) Representative multiple stretching measurements of H3.3-nucleosomes, H3.3S31ph-nucleosomes, H3.3S31A-nucleosomes, and H3.3S31E-nucleosomes. Statistical analysis of rupture forces for the outer and inner DNA wraps (**C**) and the proportion of nucleosome maintained across three stretching measurements (**D**), as shown in panels (A) and (B) (*n* = 150 for H3.3-nucleosomes, *n* = 189 for H3.3S31ph-nucleosomes, *n* = 110 for H3.3S31A-nucleosomes, *n* = 137 for H3.3S31E-nucleosomes). (**E**) Western blot analysis confirming the successful dephosphorylation of H3.3S31ph octamers by Lambda PP. (**F**) Representative multiple stretching measurements of H3.3S31ph-nucleosomes with and without Lambda PP. (**G**) Real-time traces of dephosphorylation processes for H3.3S31ph-nucleosomes. Tension was first increased from 0 to 30 pN at 10 μm/s for three cycles to observe the typical stretching measurements of H3.3S31ph-nucleosomes. Afterward, the tension was held at 8.6 pN for 5 min following the injection of Lambda PP. The dephosphorylation process was observed by a 20 nm extension jump, corresponding to the disruption of outer DNA wrap of dephosphorylated H3.3S31ph-nucleosomes at 8.6 pN. The tension was then released to 0 pN, and measurements were performed up to 30 pN at 10 μm/s for three times to identify the unfolding behavior of the dephosphorylated H3.3S31ph-nucleosomes. (**H**, **I**) Representative multiple stretching measurements of the H3.3-Nuc, H3.3S31ph-Nuc, H3.3S31A-Nuc, and H3.3S31E-Nuc in the presence of FACT. Statistical analysis of rupture forces for the outer and inner DNA wraps (**J**) and the proportion of nucleosomes maintained across three stretching cycles (**K**) for H3.3-Nuc, H3.3S31ph-Nuc, H3.3S31A-Nuc, and H3.3S31E-Nuc in the presence of FACT, as shown in panels (H) and (I) (*n*= 93 for H3.3-Nuc, *n*= 173 for H3.3S31ph-Nuc, *n*= 82 for H3.3S31A-Nuc, *n* = 59 for H3.3S31E-Nuc).

Phosphorylation of the unique residue Ser31 in the N-terminus of H3.3 has been implicated in regulating the rapid transcriptional activation in macrophages and facilitating transcriptional reprogramming during the differentiation of mESCs [20, 22]. Since the residue at position 31 is located in the N-terminus of H3.3 and resides near the DNA entry-exit site of the nucleosome (Supplementary Fig. S3A), it is of great interest to investigate how phosphorylation of H3.3S31 and its mutations regulate nucleosome dynamics and the FACT’s function at the nucleosome level. To this end, we reconstituted H3.3-nucleosomes with Ser31 phosphorylation (H3.3S31ph), a point mutation at Ser31 to alanine (S31A, the corresponding residue in canonical H3), and a phosphor-mimicking mutation (S31E) *in vitro*, using the 409-bp DNA template (Supplementary Fig. S3B). These nucleosomes were subjected to single-molecule mechanical disassembly using magnetic tweezers (Fig. 3A and B). Interestingly, although the S31A and S31E mutations do not affect the rupture forces for the outer and inner wraps of H3.3-nucleosomes, the S31ph significantly reduced the difference between the rupture forces for the outer and inner wraps (Fig. 3C). Specifically, H3.3S31ph-nucleosomes exhibited a reversible two-step structural transition at an average tension of 11.3 ± 2.6 pN (mean ± SD) during repeated stretching cycles (Fig. 3C). Furthermore, neither the Ser31 mutations (S31A or S31E) nor the phosphorylation (S31ph) affected the ability of H3.3 to maintain nucleosome integrity (Fig. 3D). These results suggested that the AIG motif within the histone fold domain of H3.3 is essential for the maintenance of nucleosome integrity, while phosphorylation at Ser31 destabilizes the nucleosome by altering its mechanical properties.

To confirm that the destabilization effect on H3.3-nucleosomes is caused by Ser31 phosphorylation, we performed dephosphorylation experiments using Lambda protein phosphatase (Lambda PP). Western blot experiments confirmed the efficient dephosphorylation of H3.3S31ph by Lambda PP *in vitro* (Fig. 3E). Force-extension measurements showed that the disruption behavior of dephosphorylated H3.3S31ph-nucleosomes reverted to that of unmodified H3.3-nucleosomes (Fig. 3F and Supplementary Fig. S3C and D). Real-time monitoring of the dephosphorylation process further supported this finding. Before the addition of Lambda PP, H3.3S31ph-nucleosomes repeatedly disrupted at a tension around 11.3 pN. Upon the addition of phosphatase, a 20-nm extension jump was observed at a constant force of 8.6 pN, corresponding to the disruption of the outer DNA wrap due to dephosphorylation (Fig. 3G). Subsequent force-extension measurements on the same nucleosomes showed a disruption mode identical to that of unmodified H3.3-nucleosomes. These results confirm that Ser31 phosphorylation destabilizes H3.3-nucleosomes, while dephosphorylation restores their stability (Fig. 3G).

Next, we examined how H3.3S31 phosphorylation or H3.3S31 mutations affect the function of FACT at the nucleosome level. Strikingly, we observed that FACT cannot maintain the integrity of H3.3S31ph-nucleosomes (Fig. 3H). In the presence of FACT, H3.3S31ph-nucleosomes were disrupted at a force of 13.9 ± 3.1 pN (mean ± SD) during the first stretching cycle, with no structural transitions observed in subsequent cycles (Fig. 3J and K). This indicated that FACT could not maintain the integrity of H3.3S31ph-nucleosomes, leading to complete nucleosome depletion after the first stretching event (Fig. 3H). In contrast, FACT maintained a stable nucleosome state for both H3.3S31A and H3.3S31E nucleosomes, similar to WT H3.3-nucleosomes (Fig. 3I). For these nucleosomes, complete disruption occurred at much higher forces centered around 25.4 pN, and the force response remained consistent across repeated stretching cycles (Fig. 3I). S31A and S31E mutations do not impair FACT’s ability to maintain the integrity of H3.3-nucleosomes, whereas Ser31 phosphorylation specifically disrupts this function. In addition, the effect of H3.3S31ph on the H2A-H2B chaperone property of FACT was also investigated (Supplementary Fig. S3E–J). The results revealed that FACT cannot maintain the integrity of H3.3S31ph tetrasomes, but can effectively deposit the H2A-H2B dimers onto H3.3S31ph tetrasomes to form intact nucleosomes. In summary, our findings reveal that phosphorylation of Ser31 in H3.3 uniquely destabilizes nucleosomes and abrogates FACT’s function in maintaining nucleosome integrity. The phosphor-mimetic mutation (S31E) fails to replicate the destabilizing effects of H3.3S31ph, underscoring the specific regulatory role of Ser31 phosphorylation in modulating nucleosome dynamics.

### H3.3 forms a sturdy nucleosome state with FACT to repress the stimulation-induced transcription in macrophages

**Figure 4.**
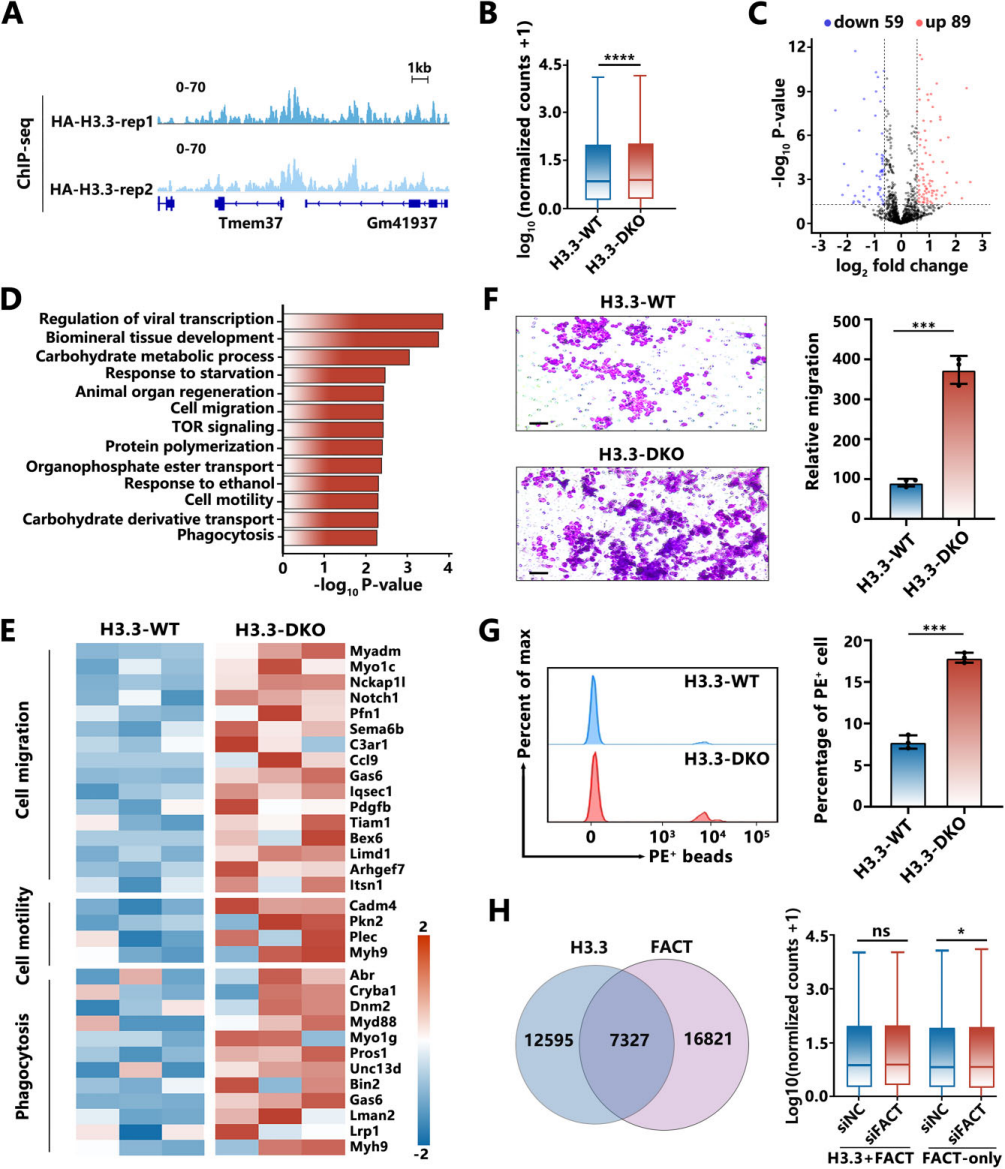
H3.3 forms a sturdy nucleosome state with FACT to repress stimulation-induced transcription in macrophages. (**A**) ChIP-seq tracks for HA-H3.3 in H3.3-WT cells at specific genomic loci, showing data for two independent replications. (**B**) Box plot analyses to compare normalized gene expression levels of H3.3-regulated genes between H3.3-WT and H3.3-DKO cells, using the Wilcoxon matched-pairs signed rank test (*P* < 2.2 × 10^−16^). ****P < .0001. (**C**) Volcano plot depicting transcriptomic changes in H3.3-regulated genes between H3.3-WT and H3.3-DKO cells. Genes with significant genes (fold change > 1.5, *P* < .05) are highlighted. (**D**) GO pathway analysis of upregulated and downregulated genes from panel (C), categorized by biological pathway using Metascape (sorted by *P*-value). (**E**) Heatmap of the typical H3.3-regulated genes in H3.3-WT and H3.3-DKO cells, associated with the GO terms “cell migration,” “cell motility,” and “phagocytosis.” Gene expression values are normalized as *Z*-scores across row-wise. (**F**) Cell migration analysis by transwell invasion assays (left) and statistical analysis (right) for H3.3-WT and H3.3-DKO cells (*n*= 3). Scale bars: 50 μm. (**G**) Phagocytosis quantification by flow cytometry in H3.3-WT and H3.3-DKO cells (left), with statistical analysis (right) (*n* = 3). The data in panels (F) and (G) were analyzed using the two-tailed *t*-test. ***P < .001. (**H**) Venn diagram analysis of ChIP-seq data for HA-H3.3 and CUT&Tag data for SPT16 in H3.3-WT cells (left). Box plots showing normalized gene expression levels for H3.3 and FACT co-regulated genes, or FACT-only regulated genes, in negative-control (siNC) and FACT-knockdown (siFACT) cells. The data were analyzed using the Wilcoxon signed-rank test, with no statistically significant difference observed in expression for H3.3 and FACT co-regulated genes between the two groups (*P* = .09657), but a significant reduction observed in expression for FACT-only regulated genes between the two groups (*P* = .01628). *P < 0.5.

Our *in vitro* investigation using a purified system revealed that the H3.3 variant can preserve nucleosome integrity and interact with FACT to form a robust nucleosome structure, which is reversed by its phosphorylation at S31. Phosphorylation of H3.3S31 has previously been shown to play an important role in facilitating rapid activation of stimulation-induced genes in macrophages [20]. To further understand how H3.3 and its phosphorylation at S31 modulate nucleosome dynamics during macrophage activation, we generated an endogenous H3.3-DKO RAW 264.7 cell line (derived from a murine leukemic monocyte/macrophage model) through CRISPR targeting of the two H3.3 genes, *H3f3a* and *H3f3b* (H3.3-DKO cells), and “rescued” the H3.3-DKO cells with HA-tagged wild-type H3.3 (H3.3-WT cells) (Supplementary Fig. S4A and B). The cells were activated by bacterial lipopolysaccharide (LPS) and interferon-gamma (IFN-γ) for 4 h to form the stimulated cells, as confirmed by western blot analysis (Supplementary Fig. S4C). Chromatin immunoprecipitation followed by sequencing (ChIP-seq) analysis of H3.3 using anti-HA antibodies was performed to define the genes directly regulated by H3.3, with typical ChIP-seq tracks for H3.3 at H3.3-regulated and non-regulated genomic regions shown. (Fig. 4A and Supplementary Fig. S4D–F). To investigate how H3.3 regulates gene expression in a stimulation-dependent manner during macrophage activation, we compared the RNA-seq data of the stimulated state for the H3.3-DKO cells and H3.3-WT cells (Supplementary Fig. S4G). By coupling the RNA-seq analysis with the H3.3 ChIP-seq analysis, we found that knockout of H3.3 statistically upregulates the H3.3-regulated genes in comparison with the H3.3-WT cells (Fig. 4B and C). GO enrichment analysis on the defined upregulated genes revealed that these genes are functionally associated with “viral transcription,” “cell migration,” “cell motility,” and “phagocytosis” (Fig. 4D and E). Hence, to further investigate the biological functions of the H3.3 variant in macrophage activation, we measured the cell migration and phagocytic ability of the stimulated cells. Remarkably, as compared to those of the H3.3-WT cells, the H3.3-DKO cells significantly enhanced the cell migration and the ability to phagocytose particles (Fig. 4F and G).

To further examine the regulation of FACT on H3.3 nucleosomes, we conducted cleavage under target and tagment (CUT&Tag) analysis of SPT16, a subunit of the FACT complex in H3.3-WT cells (Supplementary Fig. S4H). By comparing it with the genome-wide localization of H3.3, we identified the co-localized regions where both FACT and H3.3 bind (7327 regions in Fig. 4H). We then knocked down endogenous FACT expression (Supplementary Fig. S4I and J) and conducted RNA-seq analysis (Supplementary Fig. S4K). As expected, FACT knockdown statistically downregulated the FACT-bound genes without H3.3 (16 821 regions in Fig. 4H), as FACT commonly functions to facilitate chromatin transcription as it is named. However, for genes co-regulated by both FACT and H3.3 (7327 regions in Fig. 4H), the knockdown of endogenous FACT did not statistically affect the transcription of these genes. This is consistent with our *in vitro* analysis, which shows that the binding of FACT to H3.3 nucleosomes forms a stabilized nucleosome state, similar to that of H3.3-nucleosome alone.

### H3.3S31ph reverses the maintenance function of FACT at the nucleosome level and modulates stimulation-induced transcription in macrophages

**Figure 5.**
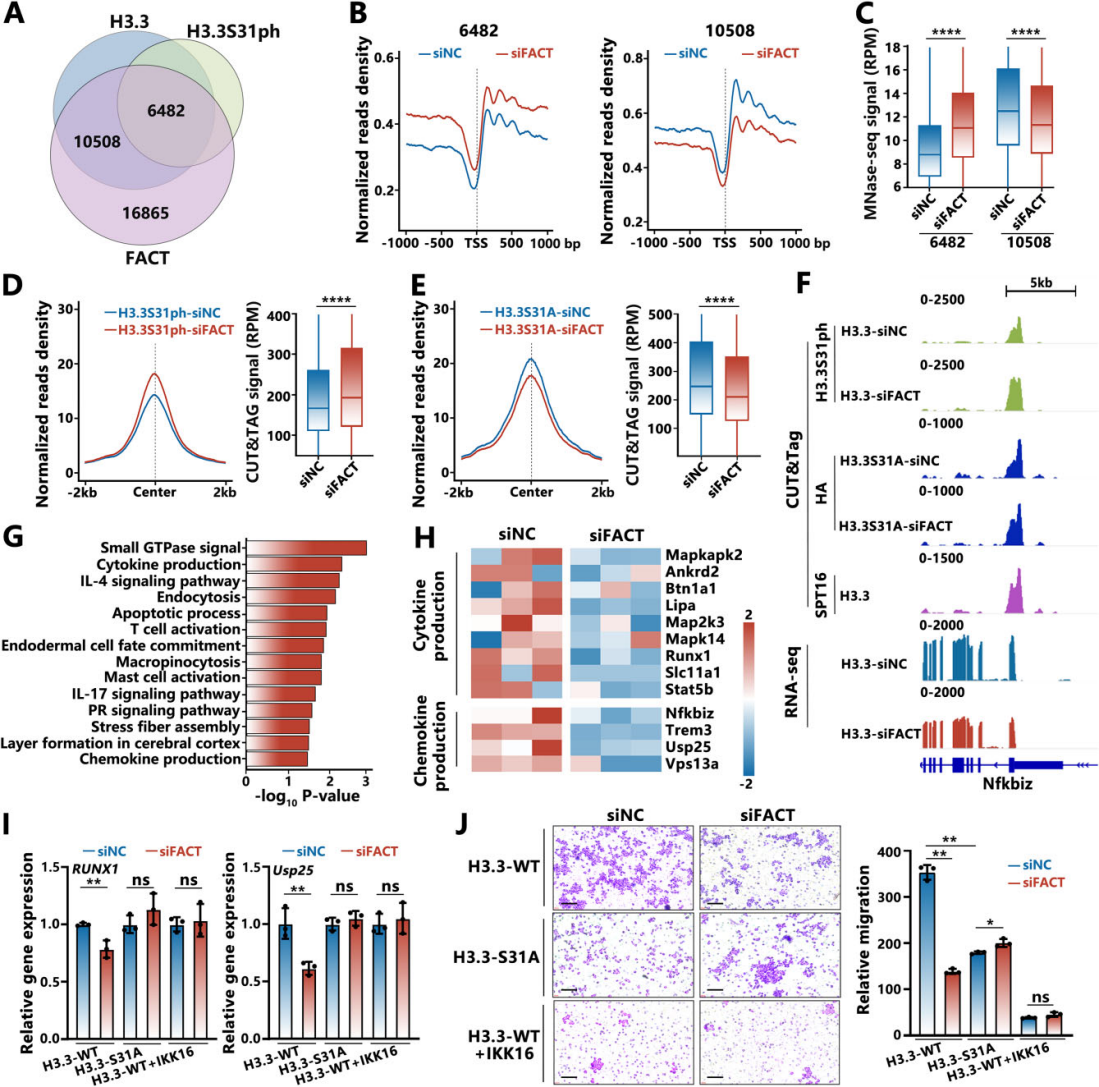
H3.3S31ph reverses the maintenance function of FACT at the nucleosome level and modulates stimulation-induced transcription in macrophages. (**A**) Venn diagram analysis of CUT&Tag data for HA-H3.3, H3.3S31ph, and SPT16 in H3.3-WT cells. (**B**) Density plot analysis of normalized nucleosome signal density for genes annotated from 6482 or 10 508 peak regions at the transcription start site (TSS). (**C**) Boxplot analysis of nucleosome signal intensity from MNase-seq data at 6482 and 10 508 peak regions. (**D**, **E**) Density plot and boxplot analysis of the read density for H3.3S31ph or H3.3S31A at 6482 peak regions in their corresponding cells. The data in panels (C), (D), and (E) were analyzed using the Wilcoxon signed-rank test. ****P < .0001. (**F**) CUT&Tag tracks (top) and RNA-seq tracks (bottom) for specific genomic loci bound by H3.3, H3.3S31A, or SPT16 in corresponding cells. (**G**) GO pathway analysis of genes annotated from 6482 peak regions, identifying differentially expressed genes in negative-control (siNC) and FACT-knockdown (siFACT) cells using DAVID (sorted by *P*-value). Significant genes (fold change > 1.5, *P* < .05) are shown. (**H**) Heatmap of genes annotated from the 6482 peak regions in negative-control (siNC) and FACT-knockdown (siFACT) cells, associated with GO terms “cytokine production” and “chemokine production.” Expression values are normalized as *Z*-scores across row-wise. RT-qPCR analysis of *RUNX1* and *Usp25* (**I**), and cell migration analysis by transwell invasion assays (left) and statistical analysis (right) (**J**) for H3.3-WT, H3.3-S31A, and H3.3-WT cells treated with IKK-16, which were transfected with negative-control short interfering RNA (siNC) or target-specific siRNA against SPT16. Scale bars: 150 μm (*n* = 3). The data in panels (I) and (J) were analyzed using the two-tailed *t*-test. *P < .05, **P < .01.

To investigate how H3.3S31ph modulates nucleosome dynamics during macrophage activation, we first examined the phosphorylation of H3.3 at Ser31 during the time-course activation of H3.3-WT cells by LPS and IFN-γ. Western blot analysis revealed the stimulation-dependent increase in H3.3S31ph (Supplementary Fig. S5A), consistent with the previous investigation [20]. To assess the genomic localization of H3.3S31ph, we performed CUT&Tag analysis of H3.3S31ph in cells stimulated for 30 min (Supplementary Fig. S5B middle). By coupling with CUT&Tag analysis of H3.3 and FACT (Supplementary Fig. S5B left and right), we identified the gene regions co-localized with H3.3S31ph and FACT (6482 regions in Fig. 5A), as well as the regions that are co-localized with unmodified H3.3 and FACT (10 508 regions in Fig. 5A) and the regions co-localized with H3 and FACT (16 865 regions in Fig. 5A).

Interestingly, the knockdown of endogenous FACT significantly increases the MNase-seq signal of 6482 gene regions that are colocalized with H3.3S31ph and FACT, but decreases the MNase-seq signal of 10 508 gene regions that are colocalized with unmodified H3.3 and FACT (Fig. 5B and C) and the MNase-seq signal of 16 865 gene regions that are colocalized with H3 and FACT (Supplementary Fig. S5C). The results were consistent with our *in vitro* analysis, which showed that FACT binding helps to maintain the integrity of unmodified H3.3-nucleosomes and canonical H3-nucleosomes, but depletes the H3.3S31ph-nucleosomes. In addition, the depletion of H3.3S31ph-nucleosome by FACT is dependent on the phosphorylation at residue Ser31. In the cells where the serine 31 of H3.3 is point-mutated to alanine (H3.3S31A-cells, Supplementary Fig. S4B), we cannot observe increased signal of H3.3S31A after knockdown of the endogenous FACT in the 6482 gene regions, as compared to that for H3.3S31ph in H3.3-WT cells (Fig. 5D and E). The typical tracks for the RNA-seq and CUT&Tag signals of FACT, H3.3S31ph, and H3.3S31A are shown for the specific gene *Nfkbiz*, which is co-regulated by H3.3S31ph and FACT in the related cells (Fig. 5F and Supplementary Fig. S5D). Both the knockdown of FACT and the S31A mutation impaired the FACT-mediated depletion of H3.3 at this region, which revealed that the depletion of H3.3 by FACT is dependent on its phosphorylation at S31. GO enrichment analysis revealed that the genes regulated by both H3.3S31ph and FACT are functionally associated with the immune response, including “cytokine production,” “chemokine production,” and “endocytosis” (Fig. 5G and H).

Additionally, H3.3S31 has been shown to be phosphorylated by the kinase IKKα at the stimulation-induced genes, which can be inhibited by the IKK inhibitor IKK-16 [20]. We examined that the addition of IKK-16 inhibited the H3.3S31ph during macrophage activation by western blot analysis (Supplementary Fig. S5E). Time-course qPCR with reverse transcription (RT-qPCR) measurement of typical genes revealed by the RNA-seq analysis, including *Runx1*, *Usp25*, and *Trem3*, showed that knockdown of FACT can impair the FACT-mediated depletion of H3.3S31ph and repress these genes, while prevention of H3.3S31 phosphorylation via the S31A mutation or the IKK-16 inhibitor fails to do so (Fig. 5I and Supplementary Fig. S5F). We also investigated migration ability of stimulated cells by using the transwell assay. The prevention of the H3.3S31 phosphorylation through S31A mutation or IKK-16 inhibitor significantly reduced the migration ability of cells, as did the knockdown of FACT (Fig. 5J). The results revealed that both the FACT complex and the phosphorylation at the H3.3S31 site are required for the FACT-mediated depletion of H3.3S31ph, which functions to regulate stimulation-induced gene transcription in macrophages.

## Discussion

**Figure 6.**
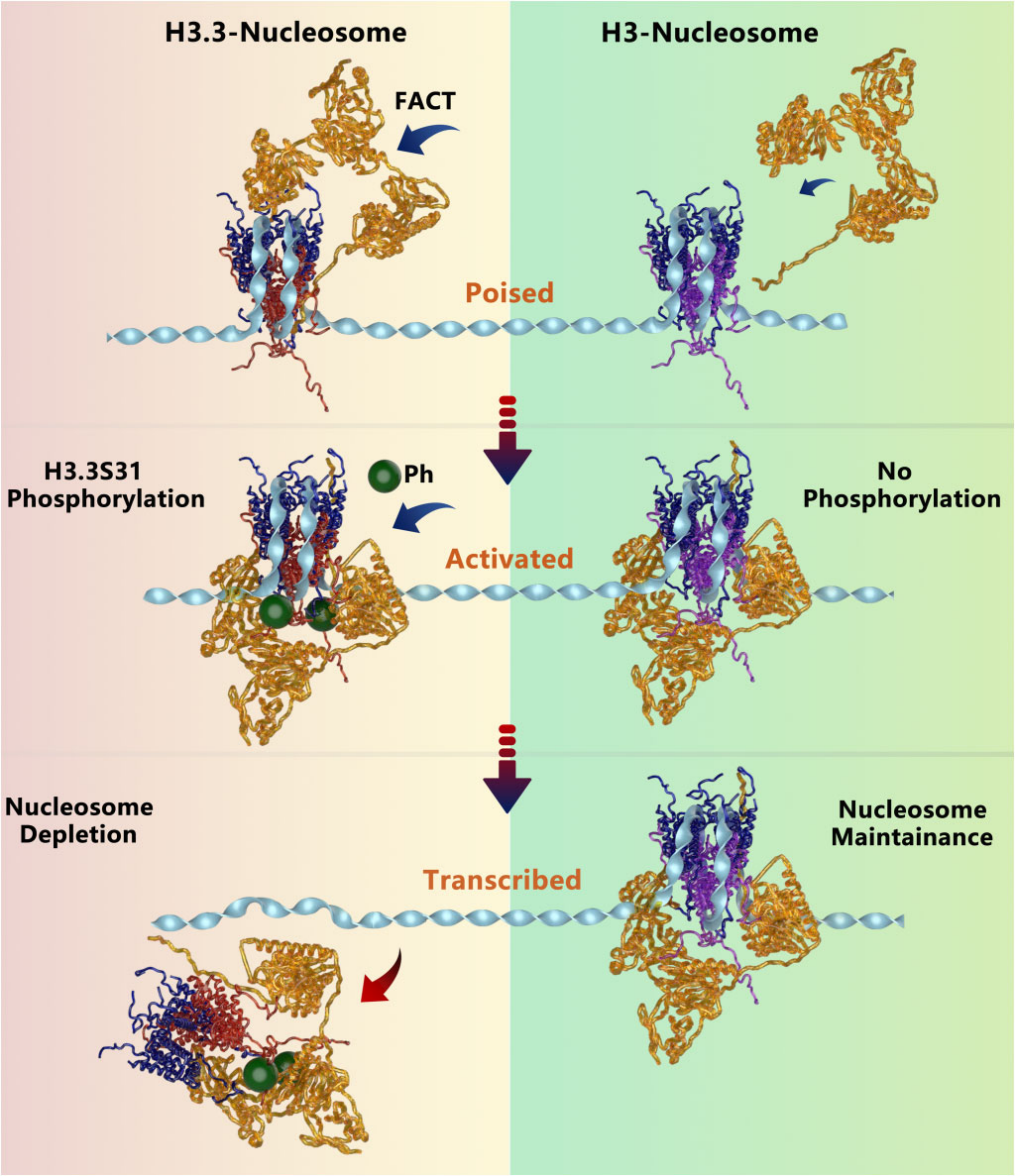
Phosphorylation of H3.3S31 serves as a molecular switch to regulate nucleosome dynamics from a stable state to an active state. Compared to histone H3, H3.3 recruits FACT complexes more efficiently, stabilizing the nucleosomes and poising transcription. Phosphorylation of the unique residue Ser31 of H3.3 triggers the FACT-mediated depletion of H3.3S31ph-nucleosomes from chromatin to activate stimulation-induced transcription in macrophages.

The histone variant H3.3, as the most conserved replacement variant for histone H3, has been observed to function as an important player in chromatin dynamics, influencing both gene expression and genome integrity. Despite its well-established importance, H3.3 appears to fulfill seemingly contradictory roles depending on its genomic context. On one hand, several studies have demonstrated that H3.3-nucleosomes are primarily associated with actively transcribed regions and more susceptible to disruption than their canonical counterparts [12, 40, 41]. On the other hand, H3.3 has also been linked to gene silencing and heterochromatin formation, particularly in repetitive genomic regions, where it regulates heterochromatin accessibility for efficient ERV repression [8, 12, 42]. The molecular mechanisms underlying these dual roles of H3.3 in euchromatin and heterochromatin remain to be elucidated. In this study, we have provided a comprehensive analysis of H3.3’s intrinsic properties and its impact on nucleosome stability and dynamics. Using *in vitro* single-molecule magnetic tweezers, we revealed that the incorporation of H3.3 significantly enhances the maintenance property of nucleosomes, without affecting their stability. Further investigation showed that the three distinct residues in the nucleosome core domain, known as the AIG recognition motif of H3.3, which determines the deposition route through direct interaction with H3.3-specific chaperones [16, 18, 19], are crucial for the maintenance property of H3.3-nucleosome integrity. Our findings also revealed that, compared to canonical H3, H3.3 promotes enhanced recruitment of the FACT complex, while substantially impairing FACT’s destabilizing effect on nucleosomes. The FACT complex, known for its role in transcription and DNA replication, has been shown by recent structural and single-molecule studies to destabilize nucleosomes and maintain the nucleosomal state [38, 43]. Interestingly, when FACT binds to H3.3-nucleosomes, it promotes a more stable, durable nucleosome state, which contrasts with its usual destabilizing effect on canonical H3-nucleosomes. A striking observation in our study is that the stability of H3.3-nucleosomes is regulated by phosphorylation at Ser31, a unique residue located in the N-terminus of H3.3. We show that phosphorylation of H3.3 at Ser31 (H3.3S31ph), but not point mutations at this site (H3.3S31A or H3.3S31E), reverses the maintenance function of FACT at the nucleosome level, switching H3.3 from a stable repressed state to an actively transcribed state in response to environmental stimuli in macrophages (Fig. 6). This functional switch underscores the importance of PTMs in modulating chromatin structure and gene transcription. Our results highlight the concept that chromatin landscape and gene transcription are not solely determined by histone variants like H3.3, but also by their unique PTMs and binding partners, which collectively shape distinct chromatin states essential for cellular function and identity. Particular histone dynamics during the transcriptional process specifically function to maintain or switch the given cellular state in a lineage. H3.3’s ability to undergo this dynamic functional switch is particularly significant for its role in maintaining cellular states during transcriptional responses. In the absence of stimulation, H3.3-nucleosomes promote a poised, maintenance-oriented chromatin state, maintaining genomic integrity and stability. Upon phosphorylation at Ser31, H3.3 switches to an active chromatin state, facilitating rapid transcriptional activation. This dual functionality of H3.3 in maintaining chromatin structure while simultaneously enabling transcriptional responsiveness positions it as a key regulator of chromatin architecture during cell differentiation and activation.

The highly conserved FACT complex has been revealed to play essential roles in almost all chromatin-related processes, including transcription, replication, and DNA damage repair. Previous investigations have shown that FACT can not only facilitate chromatin transcription as it is named, but also function to maintain genome-wide chromatin integrity [39, 44–46]. Although initially identified as a histone chaperone of the H2A-H2B dimer, FACT has been found to tether both H2A-H2B dimers and H3-H4 tetramers on DNA to form a loose nucleosome state [39, 43, 44]. In addition, dual functions of FACT on nucleosomes have been revealed by reducing the nucleosome stability and maintaining the nucleosome integrity [38]. It is of great interest to investigate how the FACT’s function is regulated by different epigenetic factors in distinct circumstances. Our previous studies have shown how modifications of H2A-H2B dimers, such as mono-ubiquitination and the variant macroH2A, modulate the function of FACT [47–49]. Specifically, we observed that H2AK119ub inhibits FACT binding, while H2BK120ub promotes FACT recruitment but impairs FACT’s destabilizing function at the nucleosome level [47, 48]. We also observed that the variant macroH2A also enhances the binding of FACT on the nucleosome, but distinctively impairs the maintenance function of FACT at the nucleosome level [49]. Here, we extend these findings by demonstrating how H3.3 modulates FACT function. We show that H3.3 enhances the binding of FACT on nucleosomes to form a sturdy nucleosome state, characterized by both high stability and high maintainability. However, upon phosphorylation of Ser31, a unique residue in the N-terminus of H3.3, the function of FACT switches, leading to the depletion of H3.3 from nucleosomes rather than its maintenance. This switch suggested a regulatory mechanism in which FACT’s role is contingent upon specific epigenetic modifications, providing new insights into how FACT functions in different chromatin contexts. Our research provides a distinct insight into the regulation of FACT’s function at the nucleosome level by different epigenetic factors, which helps to propose a unifying model for understanding how FACT functions in different chromatin circumstances.

Chromatin regulation must be tuned to enable rapid responses and facilitate gene activation to diverse environmental cues. Our findings underscore the importance of the interplay between histone variants, PTMs, and histone chaperones in orchestrating chromatin dynamics. In macrophages, H3.3S31ph acts as a critical regulatory mechanism, allowing the chromatin landscape to transition from a stable repressed state to a highly active state upon stimulation. The FACT complex ensures precise control over chromatin dynamics and the cellular response of macrophages. We showed that the selective phosphorylation at H3.3S31 appears to be a critical regulatory mechanism, allowing H3.3-nucleosomes to transition from a stable, maintenance-oriented repressed state to a dynamic, transcriptionally active state. In the resting state of macrophages, H3.3 recruits FACT to bind to the nucleosome to form a repressed, but poised state. Once the environmental stimulus comes, the H3.3S31 is phosphorylated to quickly switch the repressed state to the highly activated state for rapid response (Fig. 6). Inhibition of the H3.3S31 phosphorylation by the S31A mutation or IKK-16 inhibitor prevents this transition and significantly reduces the cell response of macrophages. This research provides mechanistic insights into how the histone variant H3.3 dynamically co-regulates with FACT by its unique phosphorylation and functions in macrophage activation. Our findings suggest that H3.3S31ph, in concert with the FACT complex, plays a central role in modulating the chromatin landscape in response to environmental signals. It would also be of great interest to determine the high-resolution structure of the complex of FACT-H3.3-nucleosome, and reveal the structural details of how H3.3 and its S31ph affect FACT’s maintenance function at the nucleosome level. All these insights could help to inform the development of targeted therapeutic strategies for diseases associated with chromatin dysfunction, such as inflammatory conditions and cancers, by manipulating the pathways that regulate H3.3 phosphorylation and FACT function.

## Supplementary Material

gkaf891_Supplemental_File

## Data Availability

The raw sequence data reported in this paper have been deposited in the Genome Sequence Archive (Genomics, Proteomics & Bioinformatics 2021) in National Genomics Data Center (Nucleic Acids Res 2022), China National Center for Bioinformation/Beijing Institute of Genomics, Chinese Academy of Sciences (GSA: CRA022284), which is publicly accessible at https://ngdc.cncb.ac.cn/gsa.
