# Supplementary material for: Phosphorylation of H3.3 at Serine 31 acts as a switch of nucleosome dynamics for transcription
Source: Nucleic Acids Res. 2025 Sep 12;53(17):gkaf891. doi: 10.1093/nar/gkaf891 (PMC12445699; doi:10.1093/nar/gkaf891)
Supplement: gkaf891_Supplemental_File [file gkaf891_supplemental_file.pdf]

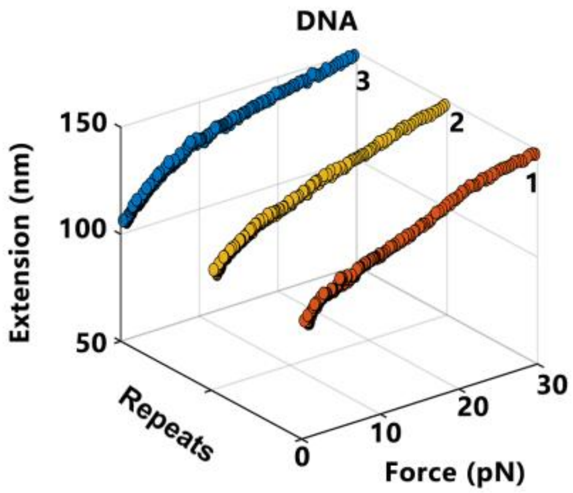

Supplementary Figure S1. Representative multiple stretching measurements of the naked DNA template, related to Figure 1.

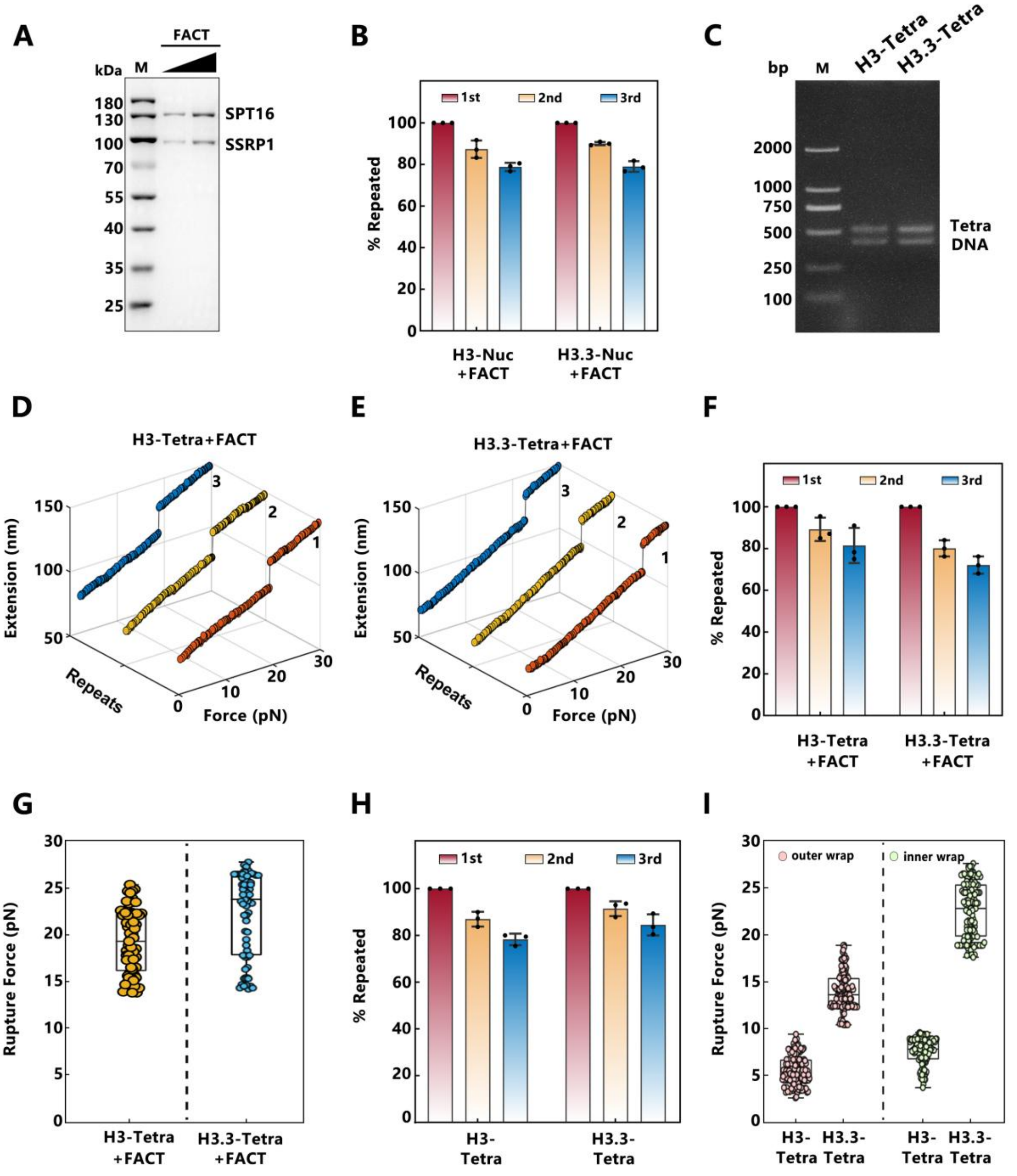

Supplementary Figure S2. FACT prefers to bind to H3.3-nucleosome to form a sturdy nucleosome state, related to Figure 2.

(A) SDS-PAGE analysis of the purified FACT complex. The data are representative of three biologically independent experiments. The first lane shows the molecular weight marker (M).

(B) The proportion of nucleosome maintained in each of the three repeated stretching measurements for H3-nucleosome (H3-Nuc) and H3.3-nucleosome (H3.3-Nuc) with FACT.

(C) 1% agarose gel electrophoresis analysis of reconstituted H3-Tetra and H3.3-Tetra for magnetic tweezer analysis. The data are representation of three biologically independent experiments. The first lane shows the molecular weight marker (M).

(D-E) Representative multiple stretching measurements of H3-tetrasome (H3-Tetra) and H3.3-tetrasome (H3.3-Tetra) with FACT, where each stretching cycle applied a

force up to 30 pN.

(F-G) Proportion of tetrasome maintained across three stretching cycles (F) and statistical analysis of rupture forces for the DNA wrap (G), as shown in panels D and E (*n* = 65 for panel D, *n* = 71 for panel E).

(H-I) Proportion of reconstituted nucleosome maintained across three stretching cycles (H) and statistical analysis of rupture forces for the outer and inner DNA wrap (I), as shown in Figure 2J (*n* = 150 for H3-Tetra, *n* = 131 for H3.3-Tetra).

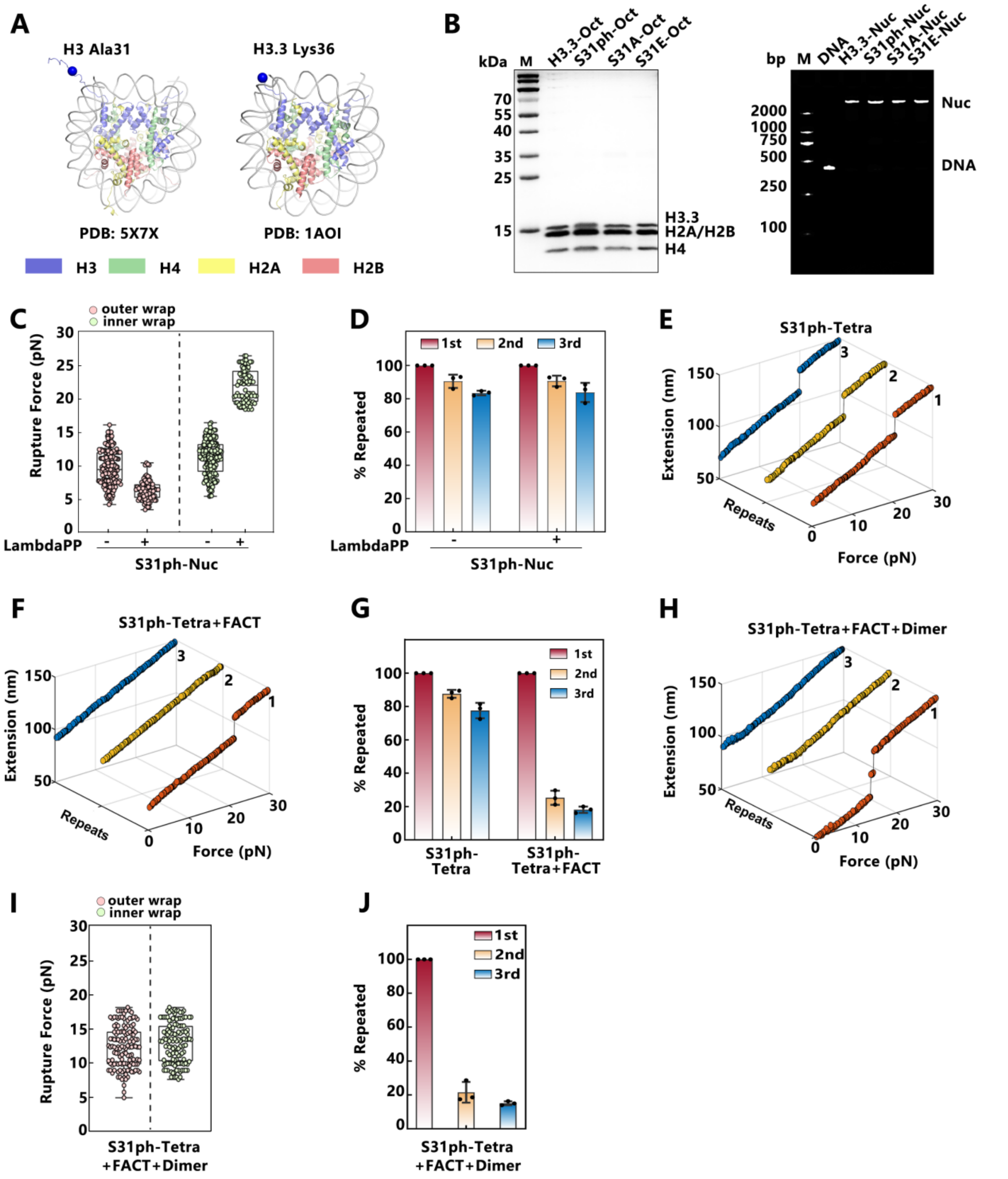

Supplementary Figure S3. Phosphorylation of H3.3 at Serine 31 (H3.3S31ph) disrupts the maintenance's function of FACT at nucleosome level, related to Figure 3.

(A) The crystal structure diagram of canonical H3- and H3.3- nucleosome, with the residues H3 Ala31 and H3.3 Lys36 highlighted.

(B) SDS-PAGE analysis (left) of the purified histone octamers with H3.3 (H3.3-Oct), H3.3S31ph (S31ph-Oct), H3.3S31A (S31A-Oct) and H3.3S31E (S31E-Oct). The first lane shows the molecular weight marker (M). 1% agarose gel electrophoresis analysis (right) of reconstituted H3.3-nucleosome (H3.3-Nuc), H3.3S31ph-nucleosome (S31ph-Nuc), H3.3S31A-nucleosome (S31A-Nuc) and H3.3S31E-nuclosome (S31E-Nuc) for magnetic tweezers analysis, using free DNA template (DNA) as control. The first lane shows the molecular weight marker (M).

(C-D) Statistical analysis of rupture forces for the outer and inner DNA wrap, as shown in Figure 3F (C) and the proportion of nucleosome maintained across three stretching cycles (D). Box plots display the median (line), interquartile range (IQR, box), data distribution (whiskers), and outliers (points beyond ± 1.5 × IQR) (n = 189 for H3.3S31ph, n = 85 for H3.3S31ph with Lambda PP).
(E-F) Representative multiple stretching measurements of H3.3S31ph-tetrasome (S31ph-Tetra) and H3.3S31ph-tetrasome with FACT.
(G) Proportion of tetrasome maintained across three stretching measurements for S31ph-Tetra and S31ph-Tetra with FACT.
(H) Representative multiple stretching measurements of S31ph-Tetra incubated with FACT and H2A-H2B dimer.
(I-J) Statistical analysis of rupture forces for the outer and inner DNA wrap (I) and proportion of nucleosome maintained across three stretching measurements (J), as shown in panel H (n = 132 for panel H).

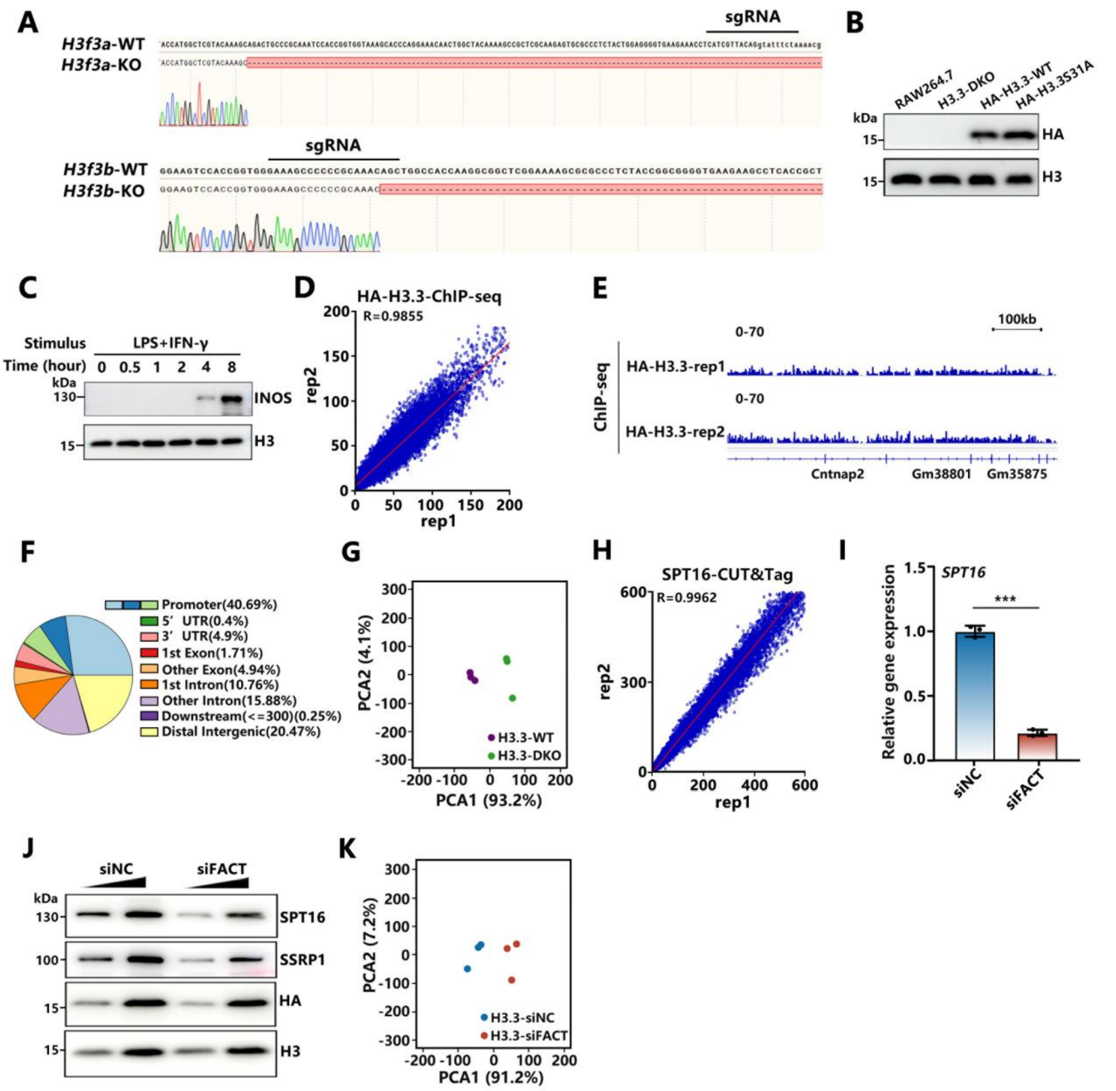

Supplementary Figure S4. H3.3 forms a sturdy nucleosome state with FACT to repress the stimulation-induced transcription in macrophage, related to Figure 4.

(A) Alignment Chart of Sequencing Results for wild-type RAW264.7 cells (RAW264.7) and *H3f3a* or *H3f3b* gene knockout cell clone.

(B) Western blot showing the protein levels of HA-H3.3 in the wild-type RAW264.7 cells (RAW264.7), H3.3 knockout cells (H3.3-DKO), cells overexpressing HA-tagged wild-type H3.3 (H3.3-WT), and cells overexpressing HA-tagged H3.3S31A (H3.3-S31A).

(C) H3.3-WT cells were stimulated with 50 ng/mL recombinant mouse IFN-γ and 100 ng/mL lipopolysaccharide at several time points, and the effects were analyzed by western blot analysis.

(D) Pearson correlation coefficient shows the correlation between two replicates of HA-H3.3-ChIP-seq data (R = 0.9855).

(E) ChIP-seq tracks for HA-H3.3 in H3.3-WT cells at genes not regulated by H3.3, showing data for two independent replications.

(F) Pie chart showing the proportion of H3.3 localization in different genomic regions.

(G) Transcriptomic principal-component analysis (PCA) of H3.3-WT cells and H3.3-DKO cells.
(H) Pearson correlation coefficient shows the correlation between two replicates of SPT16-CUT&Tag data (R= 0.9962).
(I-J) Cells were transfected with negative-control short interfering RNA (siNC) or target-specific siRNA against SPT16 (siFACT) in H3.3-WT cells, and the effects were analyzed by RT-qPCR (I) and western blot (J). The data in panel I was analyzed using the two-tailed t-test. ***$p < 0.001$.
(K) Transcriptomic principal-component analysis (PCA) of the negative-control cells (siNC) and FACT-knockdown cells (siFACT).

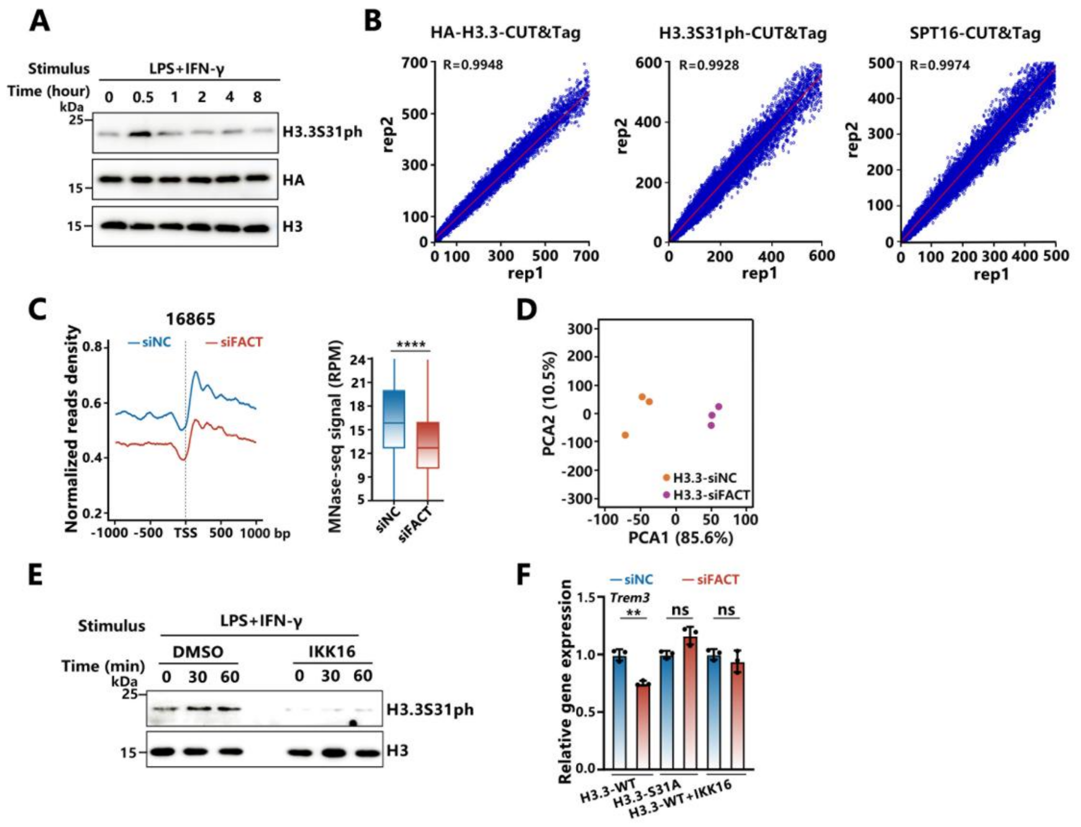

Supplementary Figure S5. H3.3S31ph reverses the maintenance's function of FACT at nucleosome level and modulates stimulation-induced transcription in macrophage, related to Figure 5.

(A) H3.3-WT cells were stimulated with 50 ng/mL recombinant mouse IFN-γ and 100 ng/mL lipopolysaccharide at several time points, and the effects were analyzed by western blot analysis.

(B) Pearson correlation coefficient shows the correlation between two replicates of HA-H3.3-CUT&Tag data, H3.3S31ph-CUT&Tag data and SPT16-CUT&Tag data. (*R*-HA = 0.9948, *R*-H3.3S31ph = 0.9928, *R*-SPT16 = 0.9974)

(C) Density plot analysis (left) of normalized nucleosome signal density for genes annotated from 16865 peak regions at the transcription start site (TSS). Boxplot analysis (right) of nucleosome signal intensity from MNase-seq data at 16865 peak regions. ****$p < 0.0001$.

(D) Transcriptomic principal-component analysis (PCA) of the negative-control cells (siNC) and FACT-knockdown cells (siFACT).

(E) Cells were treated with DMSO or IKK16 before being stimulated at three time points. The effects were analyzed by western blot analysis.

(F) RT-qPCR analysis of the gene *Trem3* in their corresponding cells. The data was analyzed using the two-tailed t test. **$p < 0.01$.
